# Identification and functional analysis of cadmium-binding protein in the visceral mass of *Crassostrea gigas*

**DOI:** 10.1038/s41598-021-90882-4

**Published:** 2021-05-28

**Authors:** Zehua Zheng, Kazuhiro Kawakami, Dingkun Zhang, Lumi Negishi, Mohamed Abomosallam, Tomiko Asakura, Koji Nagata, Michio Suzuki

**Affiliations:** 1 Department of Applied Biological Chemistry, Graduate School of Agricultural and Life Sciences, The University of Tokyo, 1-1-1 Yayoi, Bunkyo-ku, Tokyo 113-8657 Japan; 2 Frontiers Science Center for Disease-Related Molecular Network, Institutes for Systems Genetics, West China Hospital, Sichuan University, 88 Keyuan South Road, Hi-Tech Zone, Chengdu, 610041 China; 3 Institute of Molecular and Cellular Biosciences, The University of Tokyo, 1-1-1 Yayoi, Bunkyo-ku, Tokyo 113-0032 Japan; 4 Department of Toxicology, Faculty of Veterinary Medicine, Mansoura University, Elgomhouria St., Mansoura City, 35516 Egypt

**Keywords:** Biochemistry, Metals, Proteins

## Abstract

The Pacific oyster, *Crassostrea gigas*, is a traditional food worldwide. The soft body of the oyster can easily accumulate heavy metals such as cadmium (Cd). To clarify the molecular mechanism of Cd accumulation in the viscera of *C. gigas*, we identified Cd-binding proteins. 5,10,15,20-Tetraphenyl-21*H*,23*H*-porphinetetrasulfonic acid, disulfuric acid, tetrahydrate, and Cd-binding competition experiments using immobilized metal ion affinity chromatography revealed the binding of water-soluble high molecular weight proteins to Cd, including *C. gigas* protein disulfide isomerase (cgPDI). Liquid chromatography–tandem mass spectrometry (LC–MS/MS) analyses revealed two CGHC motifs in cgPDI. The binding between Cd and rcgPDI was confirmed through a Cd-binding experiment using the TPPS method. Isothermal titration calorimetry (ITC) revealed the binding of two Cd ions to one molecule of rcgPDI. Circular dichroism (CD) spectrum and tryptophan fluorescence analyses demonstrated that the rcgPDI bound to Cd. The binding markedly changed the two-dimensional or three-dimensional structures. The activity of rcgPDI measured by a PDI Activity Assay Kit was more affected by the addition of Cd than by human PDI. Immunological analyses indicated that *C. gigas* contained cgPDI at a concentration of 1.0 nmol/g (viscera wet weight)*.* The combination of ITC and quantification results revealed that Cd-binding to cgPDI accounted for 20% of the total bound Cd in the visceral mass. The findings provide new insights into the defense mechanisms of invertebrates against Cd.

## Introduction

Essential metal elements that include Na, K, Ca, Mg, Fe, as well as Cu, Zn, Ni, Se, and Mo, are classified as trace essential elements. These elements are relatively abundant in living organisms^1^. Essentiality differs between animals and plants, and non-essential elements may be classified as essential elements, depending on future research.

Essential and non-essential metal elements are required to maintain homeostasis in organisms. However, excessive levels can be toxic. Cd is a well-known toxic metal element in living organisms^2–4^. Impurities from metal refining and the combustion of fossil fuels are sources of Cd that can be released into the environment^5^. The toxicity of Cd has a great impact on the human body. One example is Itai–itai disease caused by ingestion of Cd-contaminated crops causes serious damage. This disease is one of the four major diseases caused by pollutants in Japan. Thus, Cd residues in food are an important issue in Japan. Cd is strictly regulated internationally. Limits for Cd residues in various foods include rice (approximately ≤ 0.4 ppm) and oyster (≤ 1 ppm)^6^.

Cd is a group 12 element in the same family as Zn. Cd is also a trace essential element with an electronic configuration similar to that of Zn. Therefore, Cd^2+^ can substitute for Zn^2+^ in vivo and binds to thiol groups. This can abrogate enzyme activity, which is considered to be one of the causes of Cd toxicity. Cd also inhibits the activity of proteins involved in DNA repair^7, 8^. In addition, because Cd^2+^ is classified as a soft metal by the “hard and soft acids and bases” rule, it can also replace Cu^+^ and Fe^2+^. These replacements inhibit the electron transport chain with the generation of oxygen free radicals. The consequent serious adverse effects in the body include lipid peroxidation and DNA sequence destruction^9, 10^. Furthermore, Cd^2+^ has a similar ionic radius and charge density to Ca^2+^. It has been reported that Cd^2+^ inhibits Ca^2+^-ATPase, which prevents Ca^2+^ efflux^11^.

The aforementioned variety of Cd related toxicities are reflected in the Cd defense mechanisms in some organisms^12^. One mechanism is the detoxification of Cd by the chelation of heavy metals.

Phytokeratin is a heavy metal detoxifying substance first discovered in fission yeast^13^ and later in higher plants^14^. The phytokeratin synthase activity uses glutathione as a substrate. Phytokeratin is a non-proteinaceous, low-molecular-weight peptide compound. It has a repeating structure of γ-glutamic acid-cysteine. The thiol group of the cysteine residue acts as a ligand to bond with heavy metals. One piece of evidence for the involvement of phytokeratin in Cd detoxification is the extreme Cd sensitivity of a mutant strain of *Arabidopsis thaliana* that does not synthesize phytokeratin^15^.

Metallothionein is a metal chelator in animals^16, 17^. It is a small protein composed of 60–68 amino acids that was originally discovered in horse kidney tissue^18^. It contains 20 cysteine residues and is a heat-stable protein with an amino acid sequence of C–X–C or C–X–X′–C, and no histidine or aromatic amino acids^19^. The binding between metallothionein and Cd involves 20 cysteine residues that bind to seven Cd atoms with two domains. The alpha (α) domain binds to four Cd atoms and a beta (β) domain that binds to three Cd atoms. The various functions of metallothionein include the detoxification of excess toxic metals, maintenance of homeostasis of essential biological elements such as Cu and Zn, and scavenging of radicals. A recent study reported that metallothionein is regulated in response to stress stimulation, cytokine signaling, and microbial attack by innate and adaptive immune cells^20^.

Proteomic analyses have been performed for invertebrates exposed to high concentrations of Cd. In oysters exposed to high concentrations of Cd (≥ 100 μg/L) for 96 h, a significant increase in metallothionein was evident in digestive glands. The metallothionein concentrations in gills increased to a lesser extent, and no differences were observed in adductor muscle^21^. Cd exposure elicited a rapid transcriptional response of 685 genes in oyster hemocytes, including genes mainly involved in transporter activity and metabolic processes in oysters^22^. In scallops, a significant number of genes are involved in the metabolic immune pathway and respond to a Cd stimulus^23^.

Several studies have addressed the accumulation of Cd in invertebrates, particularly mollusks. Shellfish reportedly accumulate high concentrations of Cd under normal conditions of sea water^24, 25^. In scallops, a wide range of substances with molecular weights ranging from 30,000 to 80,000 bind to Cd in the midgut line^26^. Proteins of 28, 37, and 42 kDa protein were reported to bind to heavy metals, such as Cd^27^. Squid liver accumulates 70–98% of the metal content in the entire body, especially Cd, which accumulates to a high concentration^28^. Cd-binding high molecular weight proteins have been described^29^. Other authors described Cd-binding proteins with molecular weights of 40–50 kDa^30^.

Metallothioneins have various forms ranging from small molecules to macromolecules that reportedly bind metals, such as Cd^31^. However, the structure and function of these high molecular weight Cd-binding proteins are unknown. A search failed to specifically identify naturally occurring Cd-binding proteins. Thus, it remains unclear why molluscan species such as shellfish accumulate Cd at high concentrations and whether detoxification molecules other than metallothionein are involved in Cd accumulation.

Oysters are an excellent form of nutrition as “sea milk”, which contains protein, the essential mineral Zn, Cu, Fe, Ca and other minerals, and functional ingredients^32, 33^. Pacific oysters have been used as an important protein and food source for thousands of years. Currently, oysters are globally in countries that include Japan, China, the United States, and Australia. According to a 2016 report from the Food and Agriculture Organization of the United Nations, 4.8 million tons of oysters were produced worldwide, reflecting its importance as a food source^34^. In Japan, the Pacific oyster (*Crassostrea gigas*) is most popular. Historically, it has been consumed since the Jomon period, and aquaculture production began in the Muromachi period. Currently, the Hiroshima Prefecture produces the most oysters in Japan, followed by the Miyagi and Okayama Prefectures.

*Crassostrea gigas* contains various nutrients, such as taurine and glycogen as energy sources^35^. However, toxic metals like Cd can accumulate in oysters. The mechanism of accumulation is still unknown and Cd-binding molecules have not been identified. Presently, we searched for Cd-binding substances in *C. gigas* under natural conditions.

## Materials and methods

### Animals and anatomy of tissues

**Figure 1:**
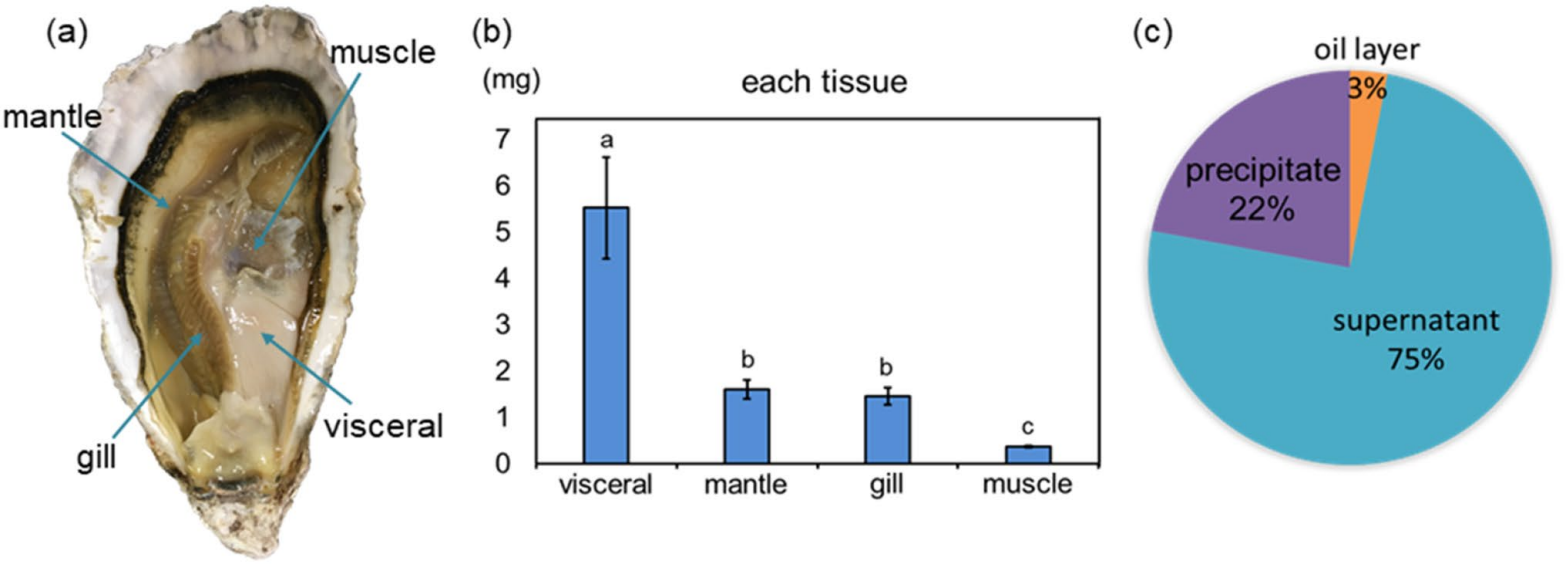
Distribution of Cd in each fraction of oyster viscera extract. (**a**) Four tissues, viscera, mantle, gills and muscle, were isolated from the dissected oysters. (**b**) The total mass of Cd contained in the visceral, mantle, gill and muscle tissues in an oyster. Error bars represent the standard deviation (n = 3); mean values with different lower-case letters (e.g., **a**–**c**) are statistically different from one another (one-way analysis of variance, ρ < 0.05). The data was analyzed using IBM SPSS software (version 18.0; SPSS Inc.). (**c**) Fan-shaped distribution diagram of Cd mass percentage in each fraction of oyster viscera extract of oil layer, supernatant and precipitate.

Three-year-old oysters (*C. gigas*) bred in Hiroshima were used. The oysters were bred for 1 day in artificial seawater to reduce stress due to environmental changes and to stabilize prior to use. The oysters were dissected into four parts (muscle, mantle, gills, and viscera) using tweezers, dissecting scissors, and a scalpel (Fig. 1a). Each tissue was frozen in liquid nitrogen, weighed, and stored at − 80 °C.

### Measurement of tissue Cd concentration

Each tissue was dried in an oven at 80 °C and 0.1 g was accurately weighed. The weighed tissues were placed in a Teflon decomposition vessel, followed by the addition of 1 mL of concentrated nitric acid. Wet ash decomposition was performed at 150 °C for 10 h. The decomposed product was diluted to 0.1 mL with 0.1 M nitric acid and measured by inductively coupled plasma-optical emission spectrometry (ICP-OES). The Cd detection wavelength was 214.438 nm. Tissue measurements were performed in tissue from three oysters.

### Extraction and measurement of Cd concentration in each layer of oyster viscera extract fraction

The oyster viscera were freeze-dried using liquid nitrogen. Extraction buffer (20 mM HEPES–NaOH, pH 7.4) was added to the ground product at a five-times weight ratio. Following thorough mixing with a vortex mixer, the samples was extracted overnight at 4 °C. After centrifuging the extract for 10 min at 4 °C and 10,000×*g*, the supernatant was collected and ultracentrifuged for 2 h at 4 °C and 100,000×*g*. The sample obtained after centrifugation comprised an oil layer, supernatant, and precipitate. The Cd concentration was measured by wet ash decomposition using the total amount of the oil layer, precipitate, and 1 mL of the supernatant.

### Post-column detection of Cd-binding substances

The supernatant of the extracted viscera was concentrated by ultrafiltration using a 0.22 µm filter. The solution was subjected to high-performance liquid chromatography (HPLC) using a YMC Pack Diol-120 gel filtration column (4.6 mm × 300 mm). Elution was performed with 0.2 µM sodium sulfate/20 mM HEPES–NaOH (pH 7.0) solution for 40 min at a flow rate of 1 mL/min. The post-column was performed with a solution containing 2 µM of 5,10,15,20-tetraphenyl-21*H*,23*H*-porphinetetrasulfonic acid (TPPS), 6 µM CdSO_4_, 50 mM HEPES–NaOH (pH 8.0), and 0.1% sodium dodecyl sulfate (SDS). A high concentration of SDS detergent can lead to protein denaturation and hinder identification of Cd-binding substances. Therefore, the minimum necessary concentration conditions for SDS were examined. We confirmed changes in the absorption spectrum when SDS was added to the post-column solution at 0.1%, 0.01%, and 0.01%. The change in absorption maximum could be suppressed more by 0.1% SDS than the other two conditions. The optimal concentration of SDS to inhibit the interaction between TPPS and proteins was investigated (Fig. S2). The column was placed in an oven at 40 °C. Protein elution was monitored by measuring the absorbance at 225 and 414 nm. Fractions were collected every 1 min without flowing the post-column solution. Each fraction was used for SDS–polyacrylamide gel electrophoresis (SDS–PAGE). The gels were stained using a Silver Quest kit (Invitrogen).

### Cd-binding competition experiment and identification of Cd-binding proteins

The extracted visceral extracts were divided into two equal volumes. One volume received 0.2 M cadmium sulfate solution to produce a final Cd concentration of 1 mM. The other volume received the same volume of ultrapure water. Both volumes were filtered using a 0.45 µm filter.

For affinity chromatography, 10 mL carrier was charged with 5 mL 0.2 M cadmium sulfate. The column was washed with 100 mL ultrapure water followed by 30 mL elution buffer, and equilibrated with 30 mL binding buffer. After loading 10 mL of the sample onto the equilibrated carrier, non-specifically adsorbed substances were washed off using 50 mL binding buffer. Substances adsorbed on the carrier were collected using 50 mL elution buffer to obtain 10 kDa species. Concentrations and desalting were performed using an ultrafiltration membrane. Ultrapure water was used for desalting. After desalting, the protein concentrations of both fractions were measured and subjected to SDS–PAGE. The gels were stained with Coomassie Brilliant Blue.

After visualization of the protein bands, the intensely stained portion confirmed from the Cd-binding competition experiment was excised and analyzed by LC–MS/MS. The gel bands or spots were excised and transferred into 1.5 mL tubes. The pieces were vortexed with 50 µL of 50% acetonitrile in 100 mM ammonium bicarbonate for decoloration and washed with 100 mM ammonium bicarbonate. Then, 100 µL acetonitrile was added and left for 15 min, with tapping every 5 min. After discarding the supernatant and drying, the pieces were incubated with 50 µL of 10 mM dithiothreitol (DTT) in 100 mM ammonium bicarbonate at 56 °C for 60 min. The supernatant was discarded and 50 µL of 55 mM iodoacetamide in 100 mM ammonium bicarbonate was added for 45 min at room temperature for alkylation. The pieces were washed with 100 mM ammonium bicarbonate for 15 min followed by 100% acetonitrile for 15 min. The process was repeated twice. After discarding the supernatant and drying, the pieces were incubated with 250 ng Trypsin Gold (Promega) and 10 µL of 50 mM ammonium bicarbonate for 6 h to digest the proteins. The supernatant was transferred to a new tube. Twenty microliters of a solution of 5% formic acid and 50% acetonitrile was added to the pieces, followed by vortexing for 20 min. The supernatant was transferred to the same tube and completely dried. Finally, 40 µL of 0.1% trifluoroacetic acid (TFA) in 2% acetonitrile was added, followed by LC–MS/MS using an Orbitrap Velos (Thermo Fisher Scientific). The LC–MS/MS data were analyzed using Proteome Discover 2.1 and the genome database of *C. gigas*^36^.

### Tissue-specific expression analysis by RT-PCR

Total RNA was extracted from each oyster visceral, mantle, gills, and muscle tissues to prepare first-strand cDNA. The PCR reaction mixture (10 µL) was adjusted according to the manufacturer’s instructions. Cycling parameters were: one cycle of 95 °C for 0.5 min, 40 cycles of 5 s at 95 °C for 20 s, and 60 °C. First-strand cDNA was used as a template for RT-PCR with PDI-5 and PDI-3 primers (Table 1). Total RNA in each tissue was estimated by PCR for actin using degenerate primers actin-F and actin-R (Table 1) designed from two conserved regions of vertebrate and invertebrate actin. The PCR cycling conditions were as follows: 35 cycles of 30 s at 94 °C (5 min for the first cycle), 30 s at 55 °C, and 2 min at 72 °C (7 min for the last cycle). PCR products were separated on a 2.0% agarose gel. Agarose gel electrophoresis results were analyzed for relative quantification using the ImageJ band density analysis.

**Table 1:** Sequences of primers used for cDNA cloning.

Primer	Sequence
cgPDI-F	CCATGTATTGCTCTCAAGGA
cgPDI-R	TCTCGCATTATCCTCATCAC
actin-F	CACAATCCTCCGCACTGGAA
actin-R	CTGATGTCAATGTCGCACGC

### Expression of *C. gigas* protein disulfide isomerase (cgPDI) in *Escherichia coli*

The cgPDI gene was amplified by PCR from the cDNA of the visceral from oyster and cloned into the *Nde*I/*EcoR*I site of pET-28a (Novagen) to obtain the expression plasmid of cgPDI with an N-terminal 6 × His-tag, which was cleaved by thrombin. The recombinant protein was overexpressed in *E. coli* BL21 (DE3). A single colony was applied to a tube containing 50 µg/mL kanamycin sulfate in 5 mL Luria–Bertani (LB) medium containing 10 g/L tryptone, 5 g/L yeast extract, and 10 g/L NaCl. The tube was cultured with shaking at 310 K for 16 h. Then, 1 mL of the previous culture solution was added to 1 L of LB medium containing 50 µg/mL kanamycin sulfate and cultured at 310 K with shaking until the optical density at 600 nm (OD_600_) reached approximately 0.5. Expression was induced by adding 1 mL 1 M isopropyl-beta-d-thiogalactopyranoside (IPTG) and culturing in a shaker incubator at 310 K for another 2 h. Culture solution was collected by centrifugation at 2000×*g* for 5 min to remove the medium. Cells were suspended in 50 mL of PBS and centrifuged at 2000×*g* for 5 min. Suspension and centrifugation were repeated twice, and finally suspended in 100 mL of PBS for ultrasonication. The suspension was ultrasonicated on ice for 20 min and then centrifuged at 2000×*g* for 10 min to obtain the soluble fraction.

### Purification of recombinant cgPDI

The soluble fraction was applied to a Ni Sepharose column and the His-tagged protein was captured. After absorbing the His-tag fusion cgPDI, the column was washed with a buffer containing 50 mM imidazole and the protein was eluted with a buffer containing 500 mM imidazole. After elution, TEV protease was used to cleave the His-tag from recombinant cgPDI (rcgPDI). Cleavage was confirmed by SDS–PAGE analysis of each fraction.

### Binding of Cd and rcgPDI

Using the composition of the post-column solution as a reference, 50 µL each of 20 µM TPPS, 60 µM cadmium sulfate, 500 mM HEPES–NaOH (pH 8.0), and 1% SDS aqueous solution was added to the protein solution (ultrapure water was used as a control). The contents were mixed with 250 µL of double deionized water. For comparison with rcgPDI, lysozyme was used. The adjusted sample was measured from 400 to 450 nm using a spectrophotometer.

### Isothermal titration calorimetry (ITC)

The sample cell was filled with 250 µL of protein solution and a syringe was filled with 60 µL of 1 mM cadmium sulfate (dissolved in 10 mM Tris–HCl [pH 8.0]). The rcgPDI dissolved in 10 µM Tris–HCl (pH 8.0) was prepared. The concentration of rcgPDI was calculated by determining the molar extinction coefficient at an ultraviolet wavelength of 280 nm (ε = 43,890 [L/(mol cm)]) from the protein sequence. The protein concentration was adjusted to 20 µM based on the absorbance value. The temperature of the sample cell was maintained at 25 °C by heating at a rate of 10 µcal/s. The syringe was used to dispense 0.4 µL for the first titration and 2 µL for the second and subsequent drops for a total of 20 times with 120 s between drops. The data were analyzed according to a model from one set of sites provided in the Origin 7.0 software for MicroCal iTC200.

### CD and inductively coupled plasma mass spectrometry (ICP-MS) analyses using Chelex treatment

To clarify the Cd-binding of rcgPDI and detect binding of rcgPDI with Cd or other metals during protein preparation, treatment with Chelex chelating metal ion exchange resin (Bio-Rad) was performed. Approximately 5 mg of resin was used for every 100 µL of cgPDI. The resin was added to the cgPDI and stirred gently for 1 h. The cgPDI was filtered from the resin to obtain the metal-free cgPDI (cgPDI-Chelex) group.

The CD spectrum was obtained using a J-820 spectropolarimeter (Jasco). rcgPDI was used as the protein sample. The samples were divided into five groups. In the pure rcgPDI, rcgPDI was dissolved in ultrapure water. In the second group, rcgPDI + Cd, rcgPDI was dissolved in ultrapure water with the addition of Cd at a final concentration of 1 mM. In the third group, rcgPDI treated with Chelex reagent, rcgPDI was dissolved in ultrapure water treated with the Chelex treatment as described above. In the fourth group, rcgPDI treated with Chelex reagent + Cd, rcgPDI was dissolved in ultrapure water treated with the Chelex treatment as explained above, followed by addition of Cd to a final concentration of 1 mM. In the fifth group, rcgPDI + Cd treated with Chelex reagent, rcgPDI was dissolved in ultrapure water with the addition of Cd at a final concentration of 1 mM, and then treated with the Chelex treatment as described above. The rcgPDI concentration was adjusted to 0.5 mg/mL. Measurements were performed at wavelengths ranging from 200 to 250 nm. The cuvette path length was 0.1 cm, scan speed was 50 nm/min, response time was 1 s, bandwidth was 1 nm, and data pitch was 0.1 nm. All samples were scanned three times and the average was taken. Secondary structure analysis was done by the BsStSel protein secondary structure analysis program^37^.

The metal concentrations were determined by ICP-MS using the 7900/MassHunter system (Agilent). The measurement conditions are summarized in Table S2. The organic matter in each sample was decomposed using nitric acid in an open wet ashing method. The sample was placed in a glass test tube, and 1 mL of concentrated nitric acid was added. The mixture was heated at 90 °C for 1 h and then at 120 °C until the nitric acid was completely evaporated. Concentrated nitric acid (1 mL) was added again and the two heating steps were repeated. Finally, 1 mL of 30% hydrogen peroxide solution (for precision analysis) was added, heated at 90 °C for 1 h, and completely evaporated at 120 °C. Heating was performed using a model DTU-2CN aluminum block constant-temperature bath (TIETECH Co., Ltd.). The decomposition product was dissolved in 5 mL of 0.08 M nitric acid and used as the measurement sample. One hundred microliters of 1000 mg/L standard solution of each metal was mixed and scaled up to 100 mL with 0.1 M nitric acid to prepare a 1 ppm standard solution.

### Tryptophan fluorescence

Tryptophan fluorescence was measured using a model FP-6500 instrument (JASCO Corporation) and an FMM-100 3 × 3 mm quartz fluorescence microcell (JASCO Corporation). The scan speed was 1000 nm/min, response time was 0.1 s, bandwidth was 5 nm, and data pitch was 10 nm. Samples were scanned three times and the average was taken. rcgPDI was used as the protein sample. The samples were divided into three groups. The first group, pure rcgPDI, comprised rcgPDI dissolved in ultrapure water. The second group, rcgPDI + Cd, comprised rcgPDI dissolved in ultrapure water with the addition of Cd at a final concentration of 1 mM. The third group, rcgPDI + Cd treated with Chelex reagent, comprised rcgPDI dissolved in ultrapure water with the addition of Cd at a final concentration of 1 mM, followed by treatment with Chelex as described above. Cadmium sulfate was added to the Cd-added group to a final concentration of 1 mM. Equal amounts of ultrapure water were added to the Cd-free group. The protein concentration was adjusted to 0.5 mg/mL. Measurements were performed at an excitation wavelength of 280 nm and an absorption wavelength of 300–450 nm.

### PDI activity

Diluted insulin solution (50 µL) was added to each tube and 10 µL of rcgPDI (prediluted enzyme to a final concentration of 2–4 units/mL) or human PDI was dispensed into each tube. This was followed by addition of 10 µL of 1 µM, 5 µM, 10 µM, 15 µM, or 20 µM cadmium sulfate and 10 µL of DTT. Each mixture was incubated at room temperature for 30 min in the dark. Next, 10 µL Stop Reagent working solution and 10 µL of PROTEOSTAT PDI Detection Reagent working solution were dispensed into each tube. The tubes were incubated in the dark for 15 min at room temperature. The generated signal was read on a fluorescence microplate reader using an excitation setting of approximately 500 nm and an emission filter of approximately 603 nm. This experiment was performed three times and the average data were recorded.

### Production of antibody to rcgPDI

Antibody production was determined by Protein Purification Industry Co. Ltd. Rabbits were injected intradermally with 1 mg of recombinant PDI. Injections were administered every 7 days for 42 days. After the final injection, the rabbits were anesthetized and whole blood was collected to obtain anti-PDI serum. Preimmune serum was obtained from rabbits prior to injection.

### Western blot

Four sheets of filter paper were immersed in solution A (50 mM Tris, 0.05% SDS, 70% methanol), two sheets in solution B (25 mM Tris, 0.05% SDS, 20% methanol), and six sheets in solution C (25 mM Tris, 0.05% SDS, 20% methanol, 40 mM 6-aminohexanoic acid) at room temperature. The polyvinylidene fluoride (PVDF) membrane was immersed in methanol for 1 min and then in solution B. After performing SDS–PAGE, the blotting apparatus was assembled while the gel was immersed in the solution B. From the anode side of the device, filter paper soaked in solution A, filter paper soaked in solution B, PVDF membrane, gel, and filter paper soaked in solution C were assembled in this order. The uppermost layer on the cathode side was brought into contact with a filter paper dipped in liquid C and transferred to the PVDF film at a constant current of 100 mA for 1 h. After transfer, the membrane was washed with TTBS (25 mM Tris, 0.8% sodium chloride, 0.02% potassium chloride, 0.1% Tween 20) and then incubated for 1 h with a blocking agent (Dainippon Pharmaceutical Co., Ltd.). After 1 h, the membrane was washed with TTBS three times for 10 min each time, and the primary antibody (anti-PDI serum or preimmune serum) diluted 1:250,000 with a blocking agent was incubated overnight at 4 °C. The membrane was then washed with TTBS three times for 10 min each time, and a secondary antibody (horseradish peroxidase conjugated goat anti-rabbit IgG, Stressgen Bioreagents) diluted 1:10,000 with a blocking agent was incubated at 4 °C for 1 h. After washing with TTBS three times for 10 min each time, substrate (SuperSignal West Pico Chemiluminescent Substrate, Pierce) was added to the membrane and incubated. Chemiluminescence was detected using a luminol image analyzer.

To quantify the content of cgPDI and its content in oyster visceral total protein. The wet weight of oyster tissue was weighed, and the visceral protein concentration of oysters was measured by mixing the solutions according to the protocol of the kit using “Protein Assay BCA Kit” purchased from Fuji Film Wako Pure Chemical Industries and measuring the absorbance at 562 nm. Bovine serum albumin solution was appropriately diluted and measured to obtain the calibration curve.

### Statistical analysis

All data are presented as the mean ± standard deviation, and differences between means were tested using Student's *t*-test (ρ < 0.05 was considered significant) and analyzed using IBM SPSS software (version 18.0; SPSS Inc.). All experiments were independently performed in triplicate.

### Ethics declarations

There is no law or unified pain classification for invertebrates in Japan. According to the recommendations of Japanese Association of Laboratory Animal Facilities of the National University Corporations, all oysters are anesthetized by low temperature treatment before dissection.

## Results

### Cd concentration in *C. gigas* tissues

The Cd concentration in each oyster tissue was measured. The Cd content was highest in the viscera, and the accumulated amount was larger in the order of mantle, gills, and muscle (Fig. 1b). Based on the tissue weight, the concentration of Cd accumulated in the viscera and gills was high, followed by the mantle and then muscle (Fig. S1). Most Cd accumulated in the viscera, with 5.5 µg of Cd per individual. The visceral part of the oyster contains a large amount of Cd-binding substances. The amount of Cd accumulated in the viscera per tissue weight was 0.34 µg/g. This result was almost equivalent to the Cd accumulation in gills (0.37 µg/g). Thus, the gills were also likely to accumulate Cd. Since the weight of the viscera is approximately five-times that of the gills, the accumulation of Cd would be greatest in the viscera. Therefore, we decided to search for Cd-binding substances from the viscera.

### Cd concentration in each fraction of viscera extracts

Viscera tissue was homogenized to extract the substances using a buffer. After extraction, the solution was ultracentrifuged to obtain an oil layer, water supernatant, and precipitate. The Cd concentration was measured in each by ICP-OES to determine the fraction of the Cd-binding substances. The oil layer contained 3% of the Cd with respect to the entire extracted fraction, the water supernatant contained 75%, and the precipitation contained 22% (Fig. 1c). The findings indicated that the water-soluble fraction, which contained proteins and sugars, also contained a large amount of the Cd-binding substance.

### Identification of Cd-binding proteins from the viscera

Free TPPS absorbed at 414 nm, while TPPS bound to Cd absorbed at 432 nm (Fig. S3a). The post-column method for detecting Cd-binding substances was performed using a gel filtration column suitable for separating substances with a wide range (low to high) of molecular weights (Fig. S3b). TPPS in the post-column solution bound to Cd. TPPS with Cd was mixed with the solution from the separated fraction using an HPLC column. If the fraction that can bind Cd was eluted, the fraction would obtain Cd from the TPPS-Cd complex, which would shift the absorbance from 432 to 414 nm. Thus, chromatogram were examined at 225 nm to detect protein and at 414 nm to detect the binding of Cd.

**Figure 2:**
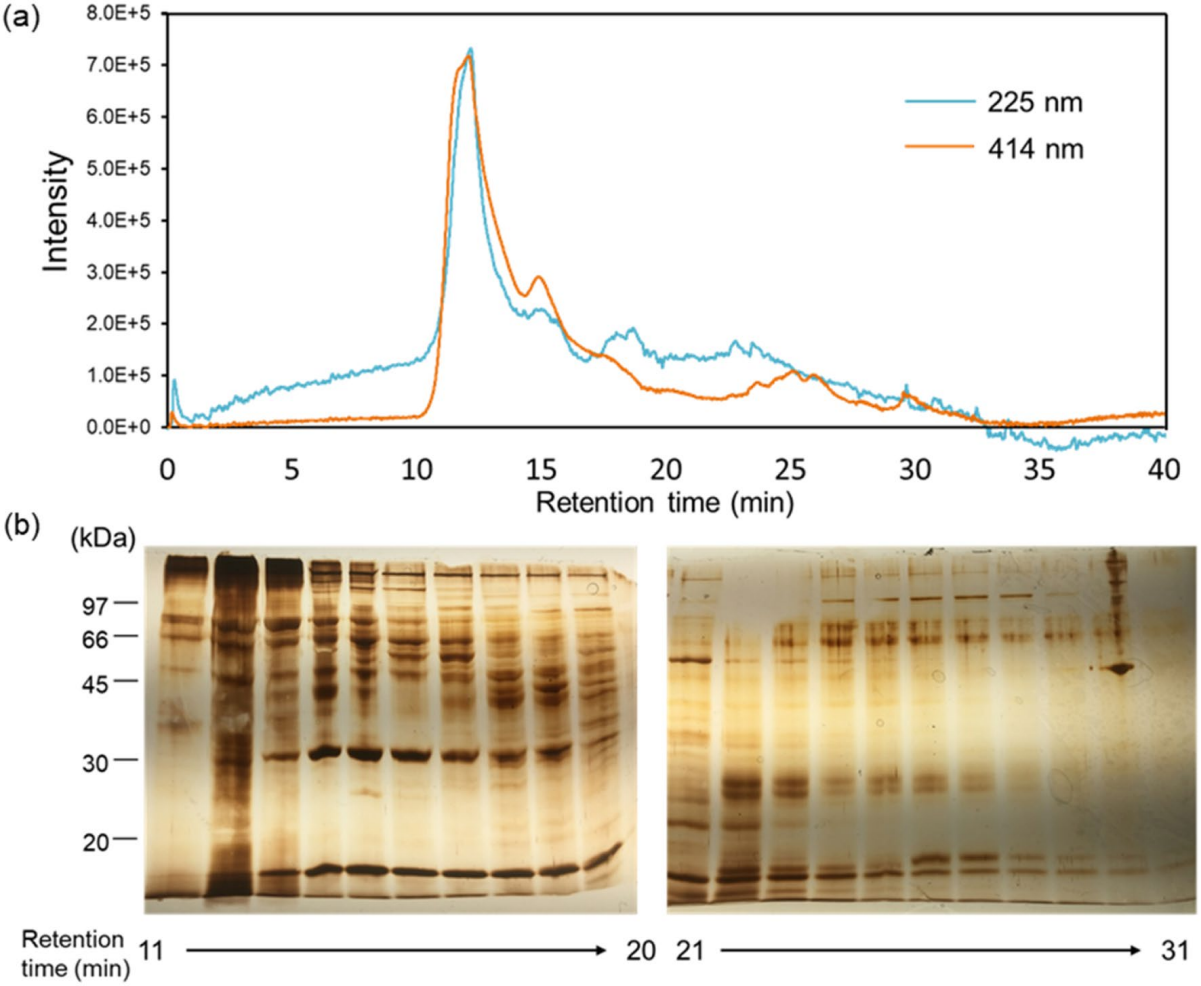
Detection and identification of Cd-binding proteins by post-column method. (**a**) The supernatant of the extracted visceral was separated by HPLC on an YMC Pack Diol-120 gel filtration column (4.6 mm × 300 mm) with post-column method. The cyan line is the signal detected at 225 nm, indicating the presence of peptides and other substances in the sample. The orange line is the signal detected at 414 nm, indicating the presence of free TPPS. (**b**) The supernatant of the extracted visceral was separated by HPLC on an YMC Pack Diol-120 gel filtration column (4.6 mm × 300 mm) without flowing the post-column solution were fractionated every 1 min, and each fraction was subjected to SDS–PAGE. From left to right are the grouping of gels cropped from two different pieces of gels, the fractions collected with retention times from 11 to 31 min.

In the chromatograms, a signal at 414 nm was observed from 11 to 18 min (Fig. 2a), which provided evidence of the presence of Cd-binding substance. Fractions were collected manually every min from to 11 to 31 min and examined by SDS–PAGE. Comparison of the gel band patterns revealed that the specific band associated with the signal at 414 nm (i.e., Cd-binding) was a high molecular weight compound of at least 30 kDa (Fig. 2b).

We prepared a chelation affinity column containing Cd to purify the Cd-binding substance. Viscera extract was applied to the affinity column. The Cd-binding molecule bound and was eluted after washing. However, as many non-specific proteins also bind to the resin of the affinity column, they had to be accounted for. An affinity chromatography competition experiment was performed. The extract from the viscera mixed with excess Cd solution was applied to the affinity column. The excess Cd inhibited specific Cd-binding in the affinity column. By comparing the changes by SDS–PAGE, the target Cd-binding substance was identified.

**Figure 3:**
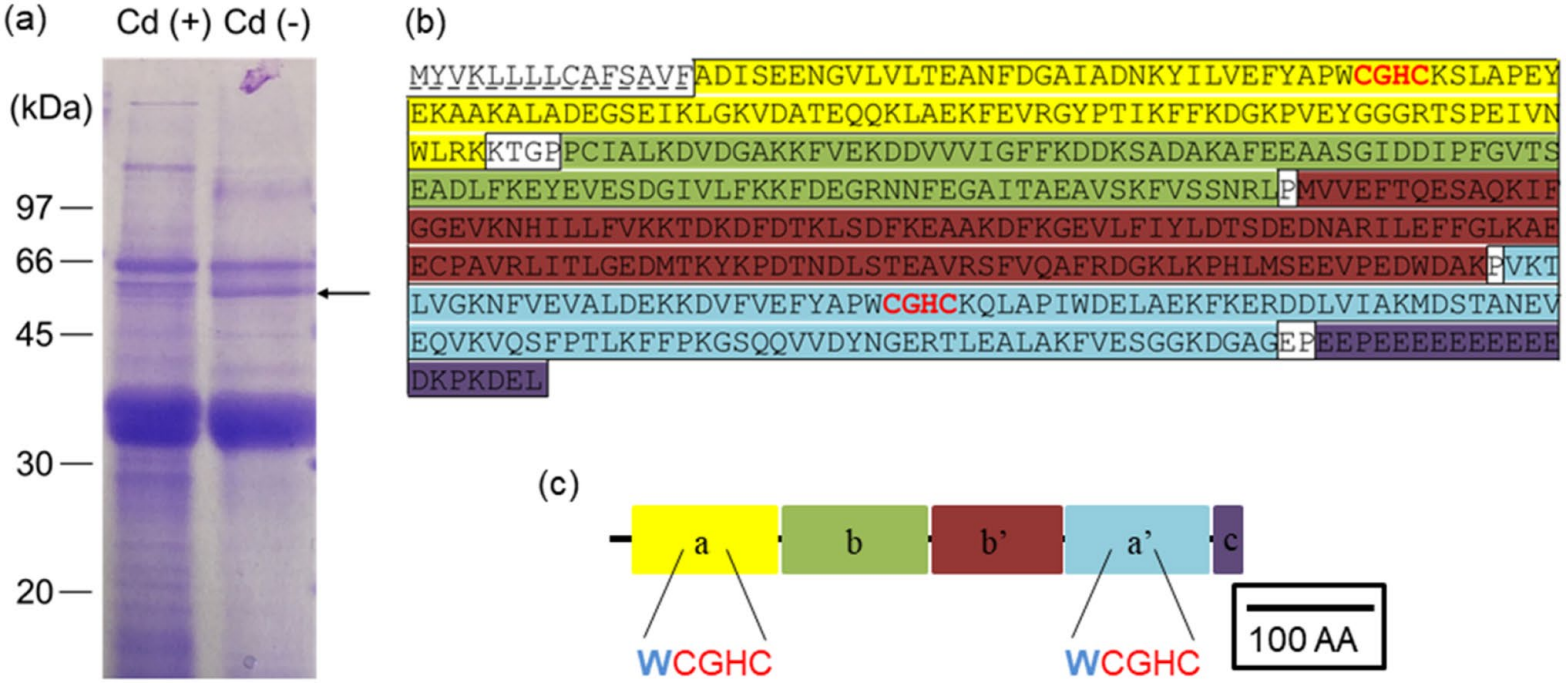
Identification of Cd-binding proteins by Cd binding competition experiment. (**a**) Cd binding competition experiment was conducted on the supernatant of the extracted visceral of oyster with and without addition of Cd. Cd (+): added with Cd. Cd (−): without Cd. Arrow: target band with high reproducibility. (**b**) The amino acid sequence of PDI. The underlines showed the signal peptide. The bold characters showed two structures of the C–X–X′–C motif as active sites. The framed areas are the five domains **a**, **b**, **b′**, **a′**, and **c** of ER_PDI_fam super family, which are also shown in (**c**). The presence of tryptophan immediately before the two CGHC sequences was shown.

Fractions with and without the addition of Cd to viscera extract were analyzed by affinity chromatography with non-specifically adsorbed substances removed. The remaining substances were then eluted and collected. Both fractions were concentrated and desalted with a 10 kDa ultrafiltration membrane and analyzed by SDS–PAGE (Fig. 3a). Although some bands disappeared with the addition of Cd, numerous repetitions of the experiment established that a band of approximately 55 kDa disappeared when Cd was present (see arrow in Fig. 3a).

In-gel digestion of this band was performed for LC–MS/MS analysis. The LC–MS/MS results are presented in Table S1. The top ten proteins with high scores are shown. We focused on CGI_10026048, which was highly reproducible and which consistently displayed the same molecular weight. The results of alignment with PDIs of another bivalve (scallop), mammalian (bovine, human), plant (soybean), nematode, and fly are shown in Fig. S4). A BLAST search for proteins implicated that cgPDI as a Cd-binding substance.

PDI has five domains (a, b, b′, a′, and c; Fig. 3b). The a, a′, b, and b′ domains include a thioredoxin-like structure. In particular, the a and a' domains have an active site with the sequence Cys–Gly–His–Cys (Fig. 3c). CgPDI has two structures having the C–X–X′–C motif, which is a characteristic sequence of metallothionein. Thus, cgPDI is likely to bind to Cd.

### Tissue-specific gene expression analysis by RT-PCR

**Figure 4:**
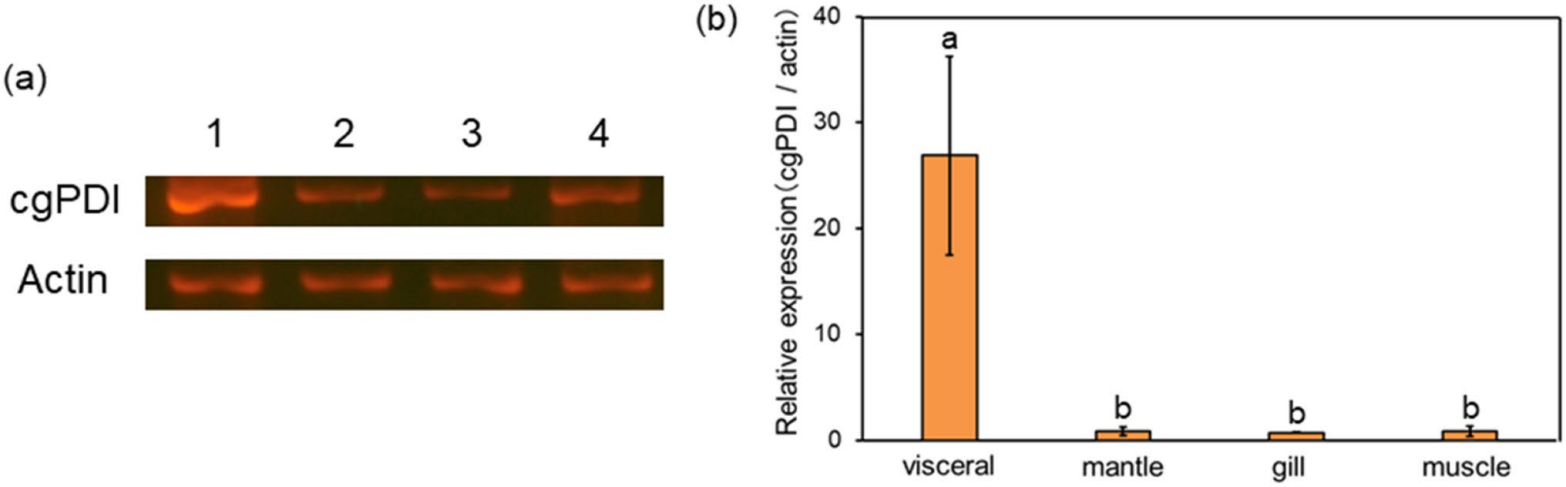
Tissue-specific expression of cgPDI by RT-PCR analysis. (**a**) cDNAs prepared from the visceral (lane 1), the mantle (lane 2), the gill (lane 3), the muscle (lane 4) were separately subjected to RT-PCR, and the products were analyzed on an agarose gel. The sequences of primers were described in Table 1. Actin as control. (**b**) Relative expression analyzed for relative quantification using ImageJ band density analysis. Error bars represent the standard deviation (n = 6); mean values with different lower-case letters (e.g., **a**,**b**) are statistically different from one another (one-way analysis of variance, ρ < 0.05). The data was analyzed using IBM SPSS software (version 18.0; SPSS Inc.).

To confirm the gene expression specificity of cgPDI, the RNA expression level of each tissue of oyster cgPDI was confirmed and a relative quantification analysis was performed. The results of cgPDI expression analysis are shown in Fig. 4a. The amount of cgPDI RNA expression at each site was determined. Strong expression of cgPDI was confirmed only in the viscera of *C. gigas* (Fig. 4b).

### Expression and purification of rcgPDI

The cgPDI was implicated in the binding of Cd. Since a large amount of cgPDI was required for further analysis, we attempted to construct rcgPDI using an *E. coli* BL21 (DE3) expression system. Bacteria were successfully transformed with a plasmid harboring the gene for rcgPDI, and the protein was expressed. The same operation was performed using an empty vector of pET28-a (+) that lacked the gene for rcgPDI (Fig. S5a). Transformants with the empty vector displayed a specific band of approximately 60 kDa.

For large-scale purification of rcgPDI, the soluble fraction of *E. coli* cell lysate was subjected to Ni-affinity chromatography. To cleave the His-tag added to rcgPDI, the fraction of the elution buffer was desalted and concentrated, and then treated with TEV protease to cleave the His-tag. After cleavage, the reaction solution was subjected to Ni-affinity chromatography. SDS–PAGE of each fraction (Fig. S5b) revealed only a band of approximately 55 kDa appeared, indicating that rcgPDI was purified. Cleavage of the His-tag of rcgPDI was also confirmed. The rcgPDI used in subsequent experiments was purified under these conditions. The purified rcgPDI was analyzed by ICP-MS to measure intact metal elements after expression in *E. coli*. There were no significant differences in Mg (*m*/*z* 24), Ca (*m*/*z* 44), Fe (*m*/*z* 56), Cu (*m*/*z* 208), Zn (*m*/*z* 66), and Cd (*m*/*z* 111) between the cgPDI obtained before and after treatment with chelating reagent. These findings suggested that there was no metal binding during the cgPDI production process (Fig. S9).

### Binding of Cd and rcgPDI using TPPS method

**Figure 5:**
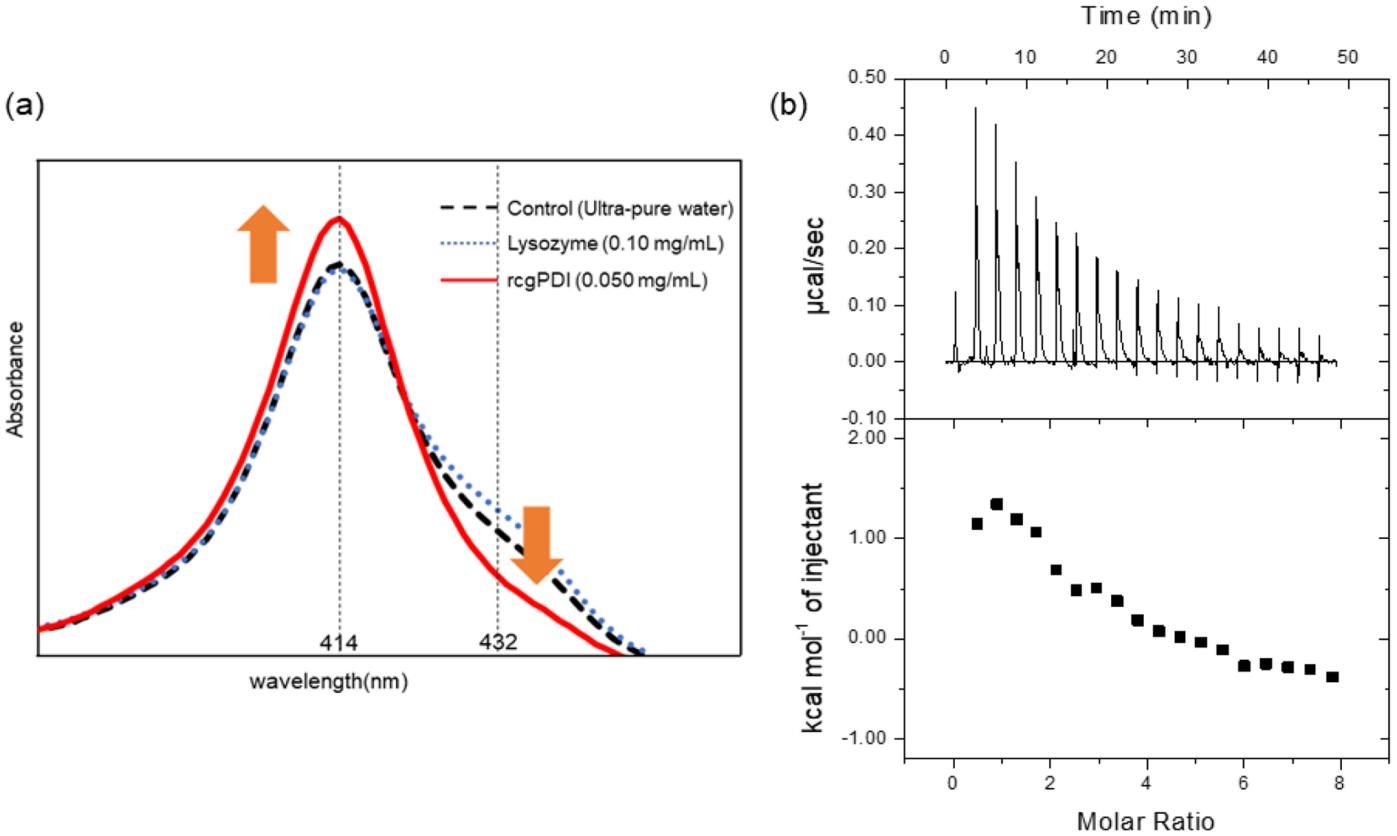
Cd-binding experiment and isothermal titration calorimetry (ITC) using rcgPDI. (**a**) Binding experiment of Cd and rcgPDI. UV/Vis absorbance spectra of the changes in absorbance at 225 nm and 414 nm wavelengths of each sample with post-column solution. Long dotted line: ultrapure water + post column solution, the dotted line: 0.1 mg/ml lysozyme + post column solution, the solid line: 0.05 mg/ml rcgPDI + post column solution. (**b**) Isothermal titration calorimetry data of rcgPDI with Cd. The upper panels show raw titration data; the lower panels are the integrated and dilution corrected peak areas of the titration data. For the titrations rcgPDI was at 20 μM, and the ligands were at 1 mM. The data were analyzed according to a model from one set of sites provided in the Origin version 7.0 software for MicroCal iTC200.

To confirm that the purified cgPDI actually binds to Cd, a Cd-binding experiment was performed. Using the composition of TPPS solution as a reference for the post-column method, a protein was added to the TPPS solution and spectrophotometry was used to detect the absorption maximum wavelength of free TPPS at 414 nm and the absorption maximum wavelength of the TPPS-Cd complex at 432 nm. Water and lysozyme comprised the negative controls. The choice of lysozyme as a negative control reflected its lack of metal binding activity and the similar molecular weight to that of cgPDI. We observed that rcgPDI bound to Cd, evident by the increased absorbance at 414 nm, which is the absorption maximum of free TPPS, and the absorbance at 432 nm, which is the absorption maximum of TPPS-Cd. These results indicated that rcgPDI was a Cd-binding substance that acquired Cd from the TPPS-Cd complex (Fig. 5a).

### ITC

To measure the binding strength between purified rcgPDI and Cd, ITC was performed upon titration of Cd with rcgPDI. An endothermic reaction due to the binding of Cd was confirmed and a curve constructed (Fig. 5b). Thermodynamic parameters could be calculated from the fitting curve, which indicated that 2.3 ± 0.3 molecules of Cd bound to each molecule of rcgPDI (Table 2).

**Table 2:** Thermodynamic parameters determined by ITC^a^.

Primer	Sequence
N	2.3 ± 0.3
K (M^−1^)	1.8 × 10^5^ ± 1.7 × 10^5^
*ΔH* (kJ/mol)	1.3 ± 0.2
*ΔS* (cal/mol/deg)	28.5

^a^Best fits were obtained by employing a sequential one site mode.

### CD and ICP-MS

The addition of Cd changes the three-dimensional structure of cgPDI and the secondary structure. Therefore, the CD spectrum of the rcgPDI was measured. The measurements were performed in the absence and presence of Cd in the range of 200 nm to 250 nm. The secondary structure was estimated by measurements using a protein secondary structure analysis program based on BeStSel.

**Figure 6:**
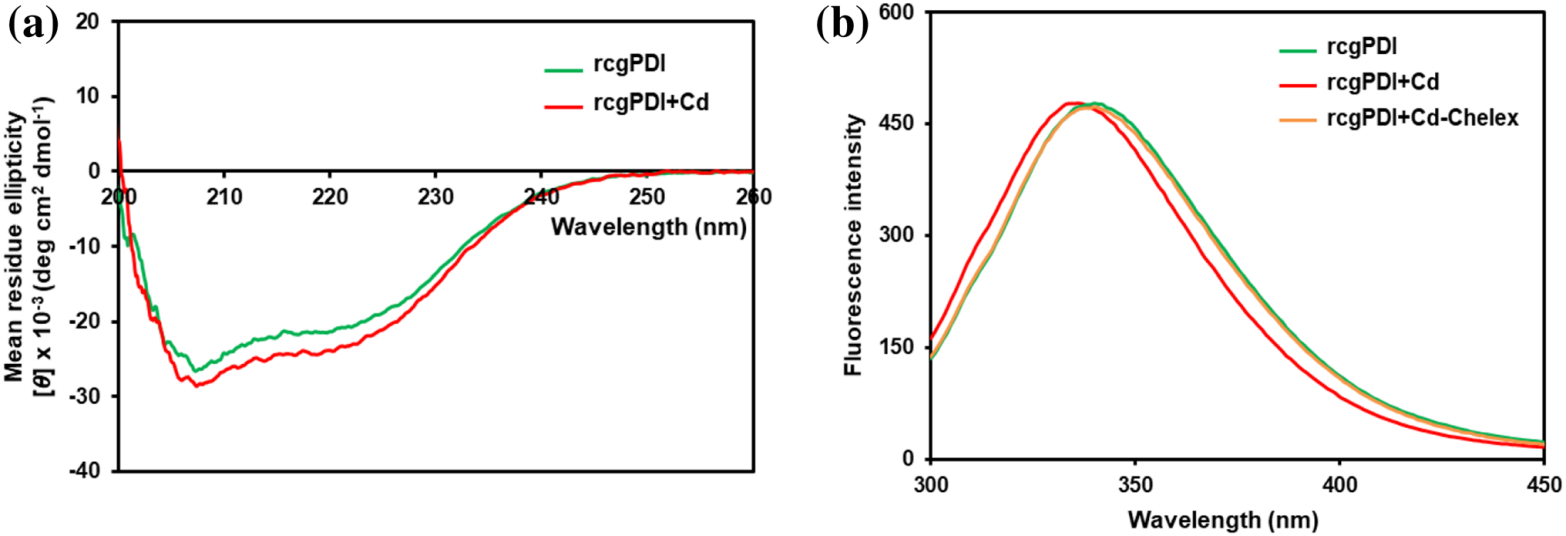
CD spectrum and tryptophan fluorescence. (**a**) UV-CD spectroscopic experiments with Cd binding rcgPDI. Far-UV-CD spectra of 0.5 mg/ml rcgPDI in a solution with and without addition of Cd at a final concentration of 1 mM. Green line: no Cd added. Red line: Cd added. *CD* circular dichroism. (**b**) Intrinsic tryptophan fluorescence emission spectra of 0.5 mg/ml rcgPDI in solutions with and without addition of Cd at a final concentration of 1 mM and 0.5 mg/ml rcgPDI after adding Cd to the solution and then subjected to chelex treatment. Excitation at 280 nm followed emission at about 335 nm (slit width for both 5 nm). An average of three fluorescence spectra of each sample were recorded at 30 °C. Green line: no Cd added. Red line: Cd added. Orange line: Cd added with after chelex treatment.

The high tension (HT) levels collected during the experiments are shown in Fig. S7. The estimated secondary structure of rcgPDI was significantly changed by the addition of Cd. The α-helix content increased from 50.9 to 57.3%, the antiparallel content decreased from 7.6 to 1.3%, and then from 7.6 to 4.3%. The “others” structure also increased from 33.9 to 37.0% (Fig. 6a) (Table 3). No significant change in the secondary structure of the rcgPDI was evident before and after treatment with chelating reagent. The secondary structure of the protein in the presence of Cd before and after treatment with chelating reagent treatment tended to be the same, indicating that the metal chelator did not change the secondary structure of the rcgPDI (Fig. S6a, b, c, g, h, i) (Table 3). After the protein was bound to Cd, the addition of the metal chelating reagent could not return the secondary structure to the natural form of rcgPDI in the absence of Cd, indicating that the structure of rcgPDI once changed is difficult to be restored. (Fig. S6d, e, f, m, n, o; Table 3).

**Table 3:** CD spectrum date analysis of rcgPDI.

	rcgPDI (%)	rcgPDI + Cd (%)	rcgPDI-chelex (%)	rcgPDI-chelex + Cd (%)	rcgPDI + Cd-chelex (%)
Helix	50.9	57.3	51.3	61.5	57.2
Antiparallel	7.6	1.3	7.6	3.1	2.8
Parallel	0.0	0.1	0.0	0.0	0.0
Turn	7.6	4.3	7.7	2.7	2.1
Others	33.9	37.0	33.4	32.7	37.8

### Measurements of tryptophan fluorescence

Tryptophan fluorescence changes as the hydrophobic environment around tryptophan changes. The rcgPDI identified as a Cd-binding substance contains two CGHC sequences. Each is a sequence characteristic of metallothionein and easily binds to Cd. In addition, tryptophan (W) is located just before the CGHC sequence. Therefore, if Cd binds to CGHC and the three-dimensional structure of cgPDI changes, tryptophan fluorescence may also change. Therefore, the tryptophan fluorescence of the rcgPDI was measured. Proteins were prepared in the absence and presence of Cd. Tryptophan fluorescence was measured for each protein at an excitation wavelength of 280 nm and an absorption wavelength range of 300–450 nm. The fluorescence spectra of rcgPDI was shiftedto 4 nm at the short range of wavelength by the addition of Cd. The finding indicated that the secondary environment of the protein was significantly changed, because the hydrophobic environment around rcgPDI tryptophan was changed (Figs. 6b and S8). After adding Cd to rcgPDI and treating with Chelex, the rcgPDI peak basically returned, indicating that the structure at WCGHC of rcgPDI recovered after Cd was removed by Chelex treatment (Figs. 6b and S8).

### PDI activity

The effect of Cd on PDI activity was also investigated using the PROTEOSTAT PDI assay kit. The kit was used to identify PDI inhibitors from compound libraries because it can quantitatively measure PDI enzyme activity in a high-throughput format. When PDI catalyzed the reduction of insulin in the presence of DTT), the insulin forms an aggregate and binds to the red fluorescent detection dye.

**Figure 7:**
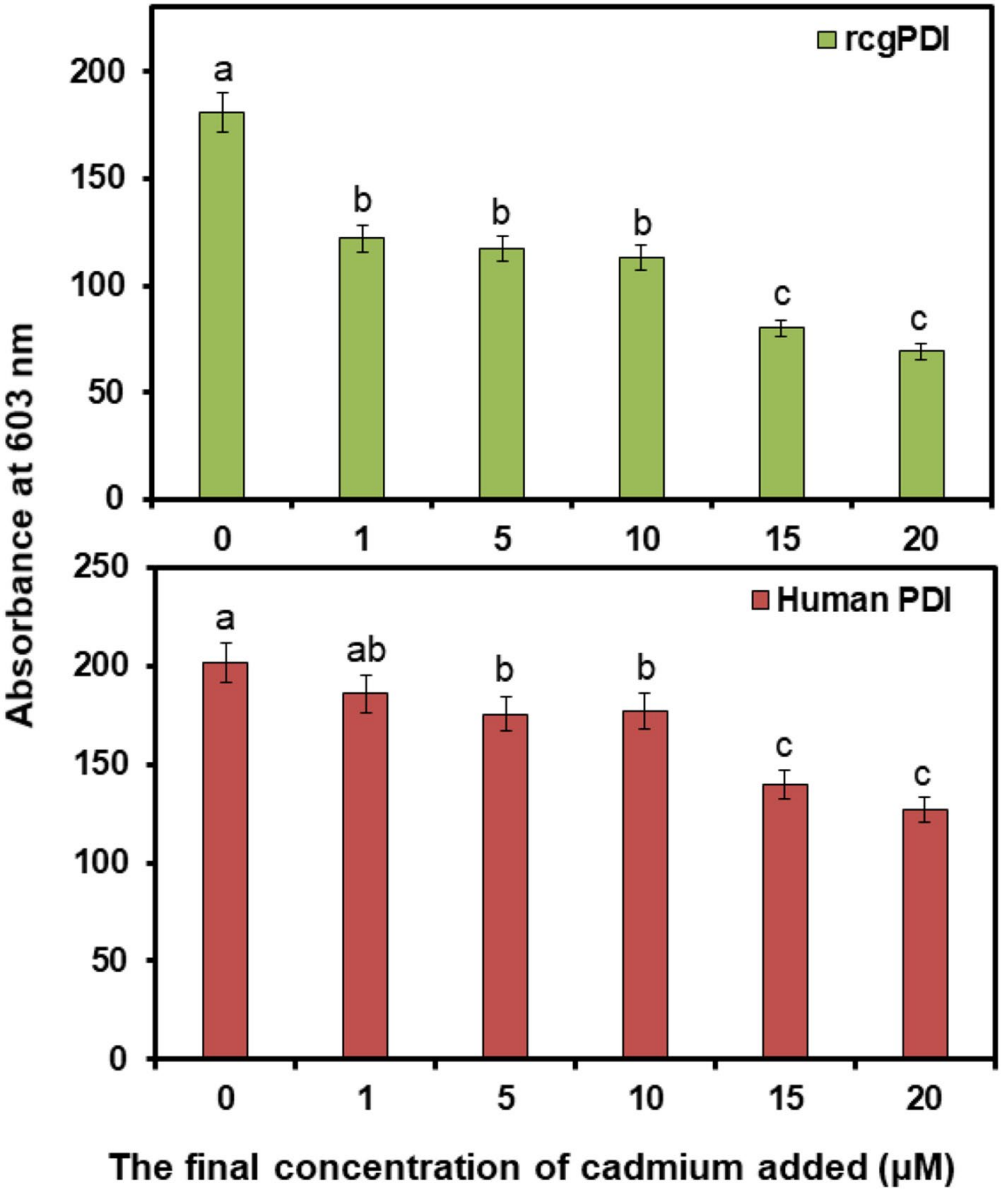
Measurement of PDI activity with Cd. The bar graph obtained from fluorescence spectrum of rcgPDI and human PDI (excitation wavelength, 500 nm, emission wavelength, 603 nm) treated with PROTEOSTAT PDI Detection Reagent in the presence of a range of concentrations of Cd (0, 5, 10, 15 and 20 µM). Green bar: rcgPDI. Red bar: human PDI. Error bars represent the standard deviation (n = 3); mean values with different lower-case letters (e.g., **a**–**c**) are statistically different from one another (one-way analysis of variance, ρ < 0.05). The data was analyzed using IBM SPSS software (version 18.0; SPSS Inc.).

In this experiment, changes in PDI activity were confirmed by adding cadmium sulfate at concentrations of 1, 5, 10, 15, and 20 µM. The human recombinant PDI enzyme (EC 5.3.4.1) in the kit was used as a control to perform the same experiment, and the change in activity was confirmed. The activities of both rcgPDI and human PDI tended to decrease with increasing Cd concentration, with a greater effect on rcgPDI than on human PDI. The findings indicate the stronger Cd-binding by rcgPDI rather than human PDI. In addition, low concentrations of Cd significantly reduced the activity of rcgPDI (Fig. 7), suggesting that rcgPDI could bind more strongly in the low Cd concentration environment.

### Western blot analysis of cgPDI in viscera

We designed and synthesized an antibody specific to cgPDI to estimate the amount of cgPDI in the viscera. Having confirmed the recognition specificity, a calibration curve was prepared using purified rcgPDI to quantify cgPDI. Western blotting was performed using 2.5, 5, 7.5, and 10 ng of rcgPDI, and 0.5 µL of oyster viscera extract (Fig. S10a). The band at approximately 55 kDa was analyzed by ImageJ image processing software to prepare a calibration curve (Fig. S10c). The PDI content in the normal state of *C. gigas* was quantified (Fig. S10b). The calculated protein weight was 13.7 µg/0.1 mg wet weight of oyster viscera, with 5.5 ng of PDI.

## Discussion

To clarify the chemical form of Cd in oysters, concentration in each tissue of *C. gigas* (visceral, gill, mantle, and muscle) was measured. Most of the Cd accumulated in the viscera (5.5 µg per oyster). The amount of Cd that accumulated in the viscera (0.34 µg/g wet weight) was almost the same as the amount of Cd that accumulated in gills (0.37 µg/g wet weight). This result was consistent with reports that heavy metals like Cd are more likely to accumulate in the viscera and gills of oysters. The digestive gland, gonad, and gills contained 1.4 ± 0.66 μg/g dry weight, 5.8 ± 2.05 μg/g dry weight, and 2.6 ± 1.87 μg/g dry weight, respectively^38^. The concentration of Cd in seawater is reportedly 0.1 ppb, as determined by ICP-MS ^39^. Presently, we calculated that the concentration of Cd in normal condition oysters was approximately 0.8 ppb in wet tissue, showing that the concentration of Cd inside the oysters is higher than that of seawater.

Ultracentrifugation of oyster extract yielded an oil layer, supernatant, and a precipitate. Compared with the oil layer and the precipitate, 75% of the extract in the supernatant contained Cd. The large amount of Cd in the water-soluble supernatant indicated that Cd^2+^ was solubilized by forming a hydrated complex or a complex with a polymer or a low-molecular weight molecule with high polarity. In vivo, Cd^2+^ is considered to be coordinated with some organic molecules. However, if a molecule with a small formation constant is coordinated, it may be replaced with a hydrated complex during the extraction process. Therefore, a post-column method for detecting Cd-binding substances using TPPS was performed to confirm whether Cd^2+^ was bound to a hydrated complex with a low or high molecular weight.

The TPPS method for Cd-binding activity has been described^40^. However, it was difficult to apply the TPPS method to the measurement of proteins. Since the planar structure of TPPS is stabilized by coordinating Cd, it is possible that it shifts to the long-wavelength side. However, when the sulfo group of TPPS and the side chain of the protein are hydrogen-bonded, TPPS conjugation is extended, shift to the longer-wavelength side is induced, and the plane is stabilized, by Cd. Therefore, we succeeded in eliminating the interaction between the sulfo group and the protein by adding SDS as a dispersant. Moreover, since SDS is used at a low concentration of 0.1%, it is considered that denaturation of the protein itself is suppressed and only the interaction is canceled.

The improved post-column method was used to search for Cd-binding substances in oysters. Cd-binding substances were evident in macromolecules > 30 kDa. When Cd in the water-soluble fraction is coordinated by a molecule with a small formation constant in vivo, it is possible that Cd could be replaced with a hydration complex during the extraction process and extracted into the water-soluble fraction. However, the high molecular weight polymer was bound to a polar substance. In a previous study, gel filtration chromatography was performed on a sample of Pacific oysters, and the Cd concentration was measured by atomic absorption spectrometry for each fraction^29^. In another reported example, gel filtration chromatography was performed on a sample from which Pacific oysters were extracted, and fractions with high Cd concentrations were purified by ion exchange chromatography. The authors reported the presence of a 40–50 kDa protein that binds to Cd in Pacific oysters^30^. The present results are consistent with these previous reports.

cgPDI was identified as a Cd-binding substance based on LC–MS/MS data. The amino acid sequences of the two active sites were completely conserved^41^. Generally, PDI is a protein that is abundant in the endoplasmic reticulum, and repair the three-dimensional structure of proteins by catalyzing the disulfide bond formation reaction. PDI is an essential enzyme for organisms^42, 43^. PDI has five domains (a, b, b′, a′, and c) ^44^. The a, a′, b, and b′ domains include a thioredoxin-like structure, and domains a and a' have an active site with the sequence Cys–Gly–His–Cys. The c domain has a sequence termed KDEL, which is an endoplasmic reticulum retention signal at the C-terminal side^45^. None of the studies have been performed in Pacific oysters to elucidate the relationship between PDI and Cd. However, comprehensive analysis of proteins in rice under Cd stress reported PDI as one of the proteins with increased expression^46^. Like the PDIs of other organisms, cgPDI has two C–X–X′–C motif structures, which are sequences characteristic of metallothionein, and therefore may be likely to bind to Cd.

CD spectrum and tryptophan fluorescence analyses were performed to determine whether the structure was changed. The CD data revealed that in the presence of Cd, rcgPDI had a higher helix ratio and β-sheet (antiparallel and parallel) ratio. The structure of cgPDI in *C. gigas* has not yet been identified, but the crystal structure of PDI in humans and some microorganisms has. PDIs in *Homo sapiens* and *Saccharomyces cerevisiae* have stable secondary structures with rich α-helix regions (30–50%) and a few β-sheets (antiparallel and parallel) regions (10–20%)^47, 48^. The estimated secondary structure of rcgPDI from the CD spectra was consistent with previous reports of PDI proteins. The CGHC sequence exists in the α-helix structure of human PDI. CGHC is also characteristic of metallothionein. It is believed that it binds to Cd and it is known to have a helical structure, while the helix of rcgPDI stabilizes the structure through the binding of Cd^49^. In addition, tryptophan fluorescence analysis revealed the shift of the rcgPDI peak to the shorter wavelength side. This shift indicates weakened hydrophobicity of tryptophan, which generates instability of the planar structure. These findings indicate that rcgPDI is bound to Cd, and that the structure is significantly changed.

In addition, when the disulfide isomerase activity of PDI was measured, the activity of human-derived PDI was not inhibited by Cd, whereas the activity of rcgPDI was inhibited by Cd in a concentration-dependent manner. These findings also suggest that cgPDI is affected by Cd in vivo. ITC was performed to calculate the binding constant of Cd and rcgPDI and the binding amount of Cd to rcgPDI. Cd and rcgPDI were endothermic. It was also shown that 2.3 molecules of Cd bind to one molecule of PDI. Because there are two active sites of CGHC, this result is valid.

We decided to use an immunological method using an antibody to calculate the actual cgPDI content of *C. gigas*. When 1 mg of the recombinant produced by *E. coli* was used to produce an antibody, a highly reactive and specific primary antibody was obtained. After confirming the specificity of the prepared antibody, western blotting was used to quantify cgPDI in *C. gigas* viscera. Quantifying cgPDI in numerous oysters indicated values of 110 ± 34.3 mg total protein and 55.0 ± 31.2 µg cgPDI per g wet weight of oyster viscera under normal conditions. Moreover, cgPDI was present in approximately 1/2000th of the total amount of protein. In the normal Pacific oyster, 1.2 µg of Cd is present per g wet weight of viscera. Thus, the viscera contains 12.5 nmol of Cd. *C. giga* contains 1.0 nmol of cgPDI per g of wet weight viscera. Considering that one molecule of cgPDI binds to 2.5 molecules of Cd, 2.5 nmol of Cd binds to cgPDI. Thus, cgPDI accounts for approximately 20% of the Cd that accumulates in *C. gigas* cultured in normal sea water. This strongly suggests that cgPDI contributes significantly to Cd accumulation in normal Pacific oysters.

## Supplementary information

Supplementary Informations.

## Data Availability

All the data is contained in the manuscript. If any more date is required, contact e-mail: amichiwo@mail.ecc.u-tokyo.ac.jp.
