# Supplementary material for: Identification and functional analysis of cadmium-binding protein in the visceral mass of Crassostrea gigas
Source: Sci Rep. 2021 May 28;11:11306. doi: 10.1038/s41598-021-90882-4 (PMC8163822; doi:10.1038/s41598-021-90882-4)
Supplement: Supplementary file 1 — Supplementary Informations. [file 41598_2021_90882_MOESM1_ESM.pdf]

# Identification and functional analysis of Cd-binding protein in the visceral of *Crassostrea gigas*

Zehua Zheng[1#], Kazuhiro Kawakami[1#], Dingkun Zhang[2], Lumi Negishi[3], Mohamed Abomosallam[4], Tomiko Asakura[1], Koji Nagata[1], Michio Suzuki[1*]

[1]Department of Applied Biological Chemistry, Graduate School of Agricultural and Life Sciences, The University of Tokyo, 1-1-1 Yayoi, Bunkyo-ku, Tokyo, 113-8657, Japan.

[2] Frontiers Science Center for Disease-related Molecular Network, Institutes for Systems Genetics, West China Hospital, Sichuan University, 88 Keyuan South Road, Hi-Tech Zone, Chengdu 610041, China.

[3]Institute of Molecular and Cellular Biosciences, The University of Tokyo, 1-1-1 Yayoi, Bunkyo-ku, Tokyo, 113-0032, Japan.

[4] Department of Toxicology, Faculty of Veterinary Medicine, Mansoura University, Elgomhouria St., Mansoura City, 35516, Egypt.

*Corresponding authors: Michio Suzuki

E-mail: amichiwo@mail.ecc.u-tokyo.ac.jp

#These authors contributed equally to this work.

**Running title:** Cd-binding protein of *Crassostrea gigas*

**Keywords:** cadmium, visceral, *Crassostrea gigas,* Cd-binding protein, protein disulfide isomerase

**Supplementary Figure legends**

Fig. S1. Cd amount in each tissue.

The mass of Cd contained per gram of oyster tissues. Error bars represent the standard deviation (n=3).

Fig. S2. Condition review of SDS concentration for TPPS method

(a) Changes in absorption spectrum of TPPS due to protein and SDS addition. Blue line: Post-column solution (free TPPS). Red line: BSA (final concentration 0.1 mg/mL) was added to post-column solution as a protein. Green line: post-column solution with BSA (final concentration 0.1 mg/mL) and SDS 1%. (b) Changes in absorption spectrum of TPPS-Cd due to SDS concentration. Blue line: post-column solution with SDS 0.1%.Red line: post-column solution with SDS 0.01%.Green line: post-column solution with SDS 0.001%.

Fig. S3. TPPS method

(a) A schematic image of TPPS reaction. TPPS-Cd showed the maximum absorbance at 432 nm. TPPS showed the maximum absorbance at 414 nm. (b) A schematic diagram shows the HPLC detection system with post-column. A novel analytical approach employing a high-performance liquid chromatography post-column method with fluorescence detection to separate and detect Cd chelators.

Fig. S4. Alignment of the sequence of cgPDI with other PDI

The clustal W computer program was used to align the amino acid sequences of the PDI. ("*" exact match, ":" group with strong similarity, "." group with weak similarity, the conserved CGHC sequence motif in the active site is boxed). XP_021350558.1, CAA89996.1, NP_776560.1, BAG16714.1, CAB07480.1 and NP_524079.1 are accession numbers of PDI from *Mizuhopecten yessoensis*, *Homo sapiens*, *Bos Taurus*, *Glycine max*, *Caenorhabditis elegans* and *Drosophila melanogaster,* respectively.

Fig. S5. Expression and purification of the recombinant cgPDI

SDS-PAGE of recombinant cgPDI. (a) pET28-a (+) empty vector soluble fraction (lane 1), pET28-a (+) PDI insert vector soluble fraction (lane 2), the fraction eluted with 500 mM imidazole in Ni affinity chromatography purification (lane 3). (b) rcgPDI before digestion of TEV-protease (lane 1), rcgPDI after digestion of TEV-protease (lane 2).

Fig. S6. CD spectrum

CD measurements with Cd binding rcgPDI. **rcgPDI**: (a), (b) and (c) are the results of three parallel experiments of measuring CD spectra of 0.5 mg/ml rcgPDI, respectively. **rcgPDI+Cd**: (d), (e) and (f) are the results of three parallel experiments of measuring CD spectra of 0.5 mg/ml rcgPDI in a solution

with addition of Cd to a final concentration of 1 mM, respectively. **rcgPDI-chelex**: (g), (h) and (i) are the results of three parallel experiments of measuring CD spectra of 0.5 mg/ml rcgPDI with chelex treatment, respectively. **rcgPDI-chelex+Cd**: (j), (k) and (l) are the results of three parallel experiments of measuring CD spectra of 0.5 mg/ml rcgPDI with chelex treatment in a solution with addition of Cd to a final concentration of 1 mM, respectively. **rcgPDI+Cd-chelex**: (m), (n) and (o) are the results of three parallel experiments of measuring CD spectra of 0.5 mg/ml rcgPDI treated with a chelex agent after the addition of Cd to a final concentration of 1 mM, respectively.

Fig. S7. HT levels

HT levels collected during the measurement of CD spectrum. (a), (b), (c), (d), (e), (f), (g), (h), (i), (j) (k), (l), (m), (n) and (o) respectively represent the HT levels collected during the measurement of CD spectrum (a), (b), (c), (d), (e), (f), (g), (h), (i), (j) (k), (l), (m), (n) and (o) in the Fig. S6.

Fig. S8. Tryptophan fluorescence

Intrinsic tryptophan fluorescence emission spectra of 0.5 mg/ml rcgPDI in solutions. Excitation at 280 nm followed emission at about 335 nm (slit width for both 5 nm). An average of three fluorescence spectra of each sample were recorded at 30°C. Green line: no Cd added. Red line: Cd added. **rcgPDI**: (a), (b) and (c) are the results of three parallel experiments of measuring intrinsic tryptophan fluorescence emission spectra of 0.5 mg/ml rcgPDI, respectively. **rcgPDI+Cd**: (d), (e) and (f) are the results of three parallel experiments of measuring intrinsic tryptophan fluorescence emission spectra of 0.5 mg/ml rcgPDI in a solution with addition of Cd at a final concentration of 1 mM, respectively. **rcgPDI+Cd-chelex**: (g), (h) and (i) are the results of three parallel experiments of measuring intrinsic tryptophan fluorescence emission spectra of 0.5 mg/ml rcgPDI treated with a chelex agent after the addition of Cd at a final concentration of 1 mM, respectively.

Fig. S9. ICP-MS

The concerntration of Mg (*m/z* 24), Ca (*m/z* 44), Fe (*m/z* 56), Cu (*m/z* 208), Zn (*m/z* 66), Cd (*m/z* 111) in rcgPDI before or after chelex treatment was measured by ICP-MS. Error bars represent the standard deviation (n=3). rcgPDI-chelex: rcgPDI after chelex treatment.

Fig. S10. Quantification of cgPDI by Western blot with anti-cgPDI.

(a) SDS-PAGE of Western blot. Standard sample 1: rcgPDI 10 ng, 2: rcgPDI 7.5 ng, 3: rcgPDI 5.0 ng, 4: rcgPDI 2.5 ng (b) SDS-PAGE of Western blot. Oyster extract (use 0.5 µl). (c) Standard curve of cgPDI by Western blotting using anti-cgPDI. Western blotting was performed using 2.5 ng, 5 ng, 7.5 ng, and 10 ng of rcgPDI, and the band around 55 kDa was image-processed by ImageJ to prepare a calibration curve.

Fig. S11. Full-length image of Western blot with anti-cgPDI.

Full-length membrane image of Western blot in Fig. S10.

Fig. S12. Full-length image of agarose gel in tissue-specific expression of cgPDI by RT-PCR analysis.

Full-length gel image in Fig. 4a.

**Supplementary Table**

Table S1. Result of LC/MS/MS analysis of the 55-kDa protein in Cd binding competition experiment. The gene (CGI_10026048) indicated by red color appeared in both measurements and the molecular weight of CGI_10026048 was matched to that of the band estimated from the SDS-PAGE.

(a) LC/MS/MS results of 1st measurement

| Accession | Sum PEP Score | Coverage | MW [kDa] | calc. pI |
|---|---|---|---|---|
| CGI_10000669 | 106.87 | 72.78 | 55.7 | 6.92 |
| CGI_10026048 | 27.97 | 26.06 | 55.5 | 4.72 |
| CGI_10011652 | 27.82 | 29.27 | 55.5 | 5.83 |
| CGI_10003521 | 27.02 | 9.91 | 323.5 | 5.77 |
| CGI_10026868 | 14.55 | 21.50 | 62.1 | 7.33 |
| CGI_10005485 | 12.37 | 21.84 | 46.6 | 6.34 |
| CGI_10013834 | 10.33 | 17.05 | 62.1 | 5.90 |
| CGI_10003464 | 9.44 | 16.89 | 42.7 | 5.67 |
| CGI_10014159 | 8.48 | 3.92 | 74.5 | 8.37 |
| CGI_10018510 | 7.93 | 20.25 | 53.3 | 6.58 |

(b) LC/MS/MS results of 2nd measurement

| Accession | Sum PEP Score | Coverage | MW [kDa] | calc. pI |
|---|---|---|---|---|
| CGI_10021481 | 95.40 | 70.00 | 39.6 | 7.03 |
| CGI_10026868 | 53.62 | 35.84 | 62.1 | 7.33 |
| CGI_10003521 | 46.98 | 9.71 | 323.5 | 5.77 |
| CGI_10019801 | 36.66 | 43.97 | 43.5 | 6.20 |
| CGI_10017668 | 29.17 | 43.51 | 43.2 | 8.18 |
| CGI_10026867 | 27.15 | 21.50 | 53.1 | 6.07 |
| CGI_10026048 | 24.07 | 28.89 | 55.5 | 4.72 |
| CGI_10027059 | 23.04 | 34.01 | 32.5 | 8.37 |
| CGI_10022007 | 20.54 | 6.47 | 211.4 | 7.06 |
| CGI_10006610 | 15.71 | 23.23 | 39.2 | 4.93 |

Table S2. ICP-MS measurement conditions

| | | |
|---|---|---|
| ICP-MS system | G8403A / G7201C C.0105 / I-AS | |
| | Torch injector inner diameter | 2.5 mm (standard) |
| | Nebulizer | Micro Mist Nebulizer (Sample tube inner diameter 0.25 mm x 21 cm) |
| | Interface | Ni made |
| | Ion lens | x type |
| Plasma conditions | RF power | 1550 W |
| | Sampling position | 10 mm |
| | Nebulizer gas | 1.05 L / min |
| Sample introduction conditions | Nebulizer pump (for drainage) | 0.15 rps |
| | Spray chamber temperature | 2 °C |
| Ion lens condition | lead-out electrode | 0 V |
| Octapole cell condition | H2 flow rate | 6 mL / min |
| | Energy Discrimination | 3 V |
| Detection conditions | Measurement element | Mg (m/z 24), Ca (m/z 44), Fe (m/z 56), Cu (m/z 63), Zn (m/z 66), Cd (m/z 111)<br>Integral for 0.30 seconds each |
| | Online internal standard element | In (m / z 115)<br>Integral for 0.05 seconds each |

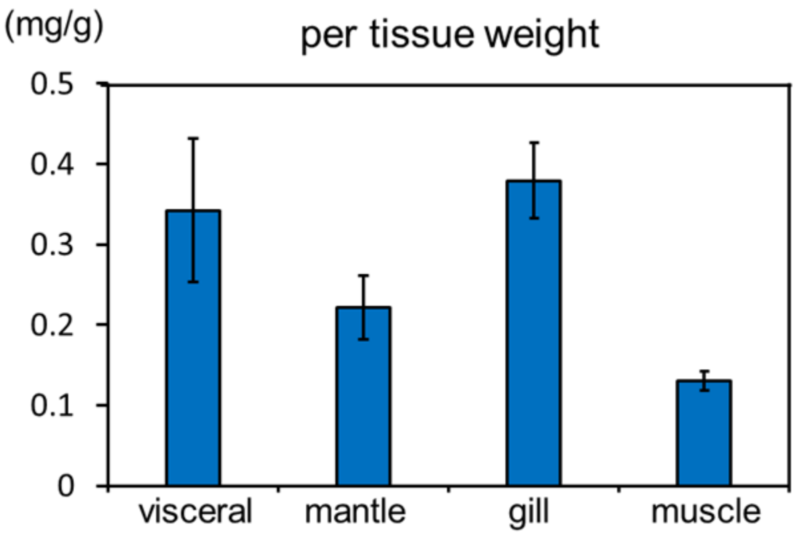
(mg/g)
per tissue weight
0.5
0.4
0.3
0.2
0.1
0
visceral
mantle
gill
muscle

**Fig. S1**

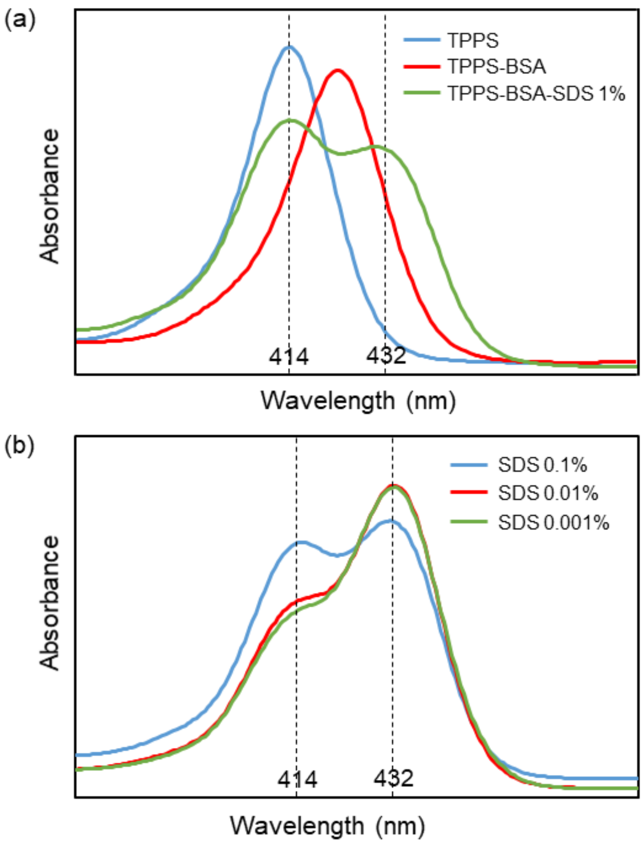
(a)
TPPS
TPPS-BSA
TPPS-BSA-SDS 1%
Absorbance
414
432
Wavelength (nm)
(b)
SDS 0.1%
SDS 0.01%
SDS 0.001%
Absorbance
414
432
Wavelength (nm)

**Fig. S2**

(a)

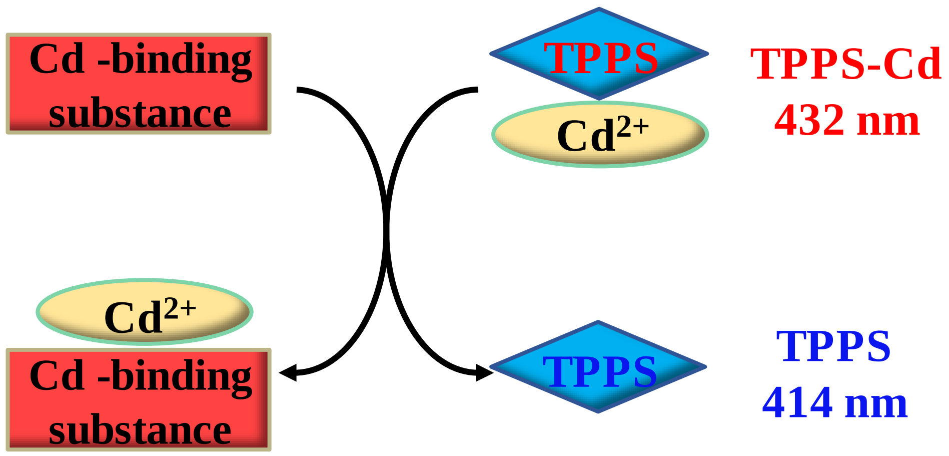
Cd -binding substance
TPPS
Cd2+
TPPS-Cd
432 nm
Cd2+
Cd -binding substance
TPPS
TPPS
414 nm

(b)

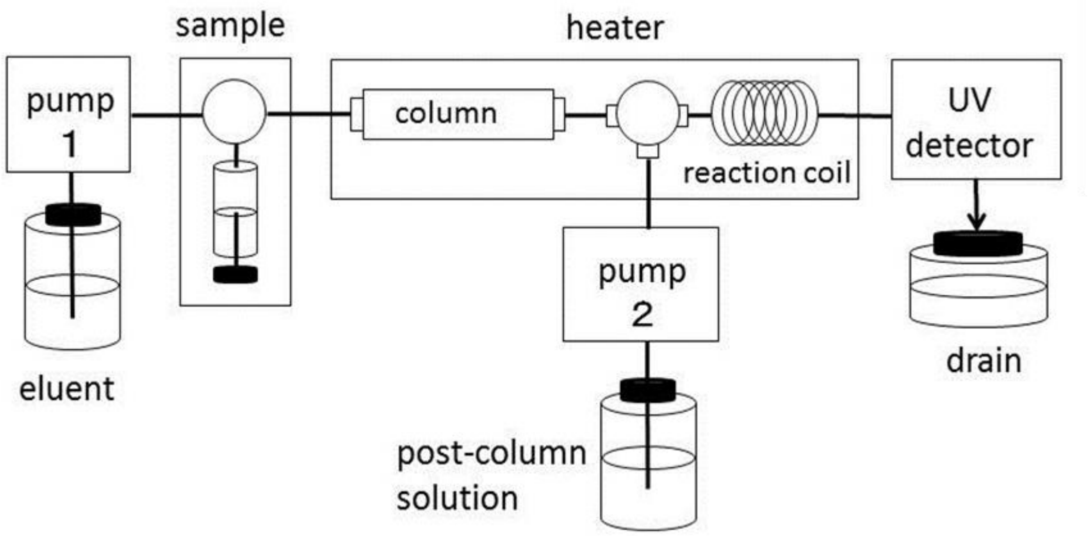
sample
heater
pump
1
column
reaction coil
UV
detector
pump
2
eluent
drain
post-column
solution

**Fig. S3**

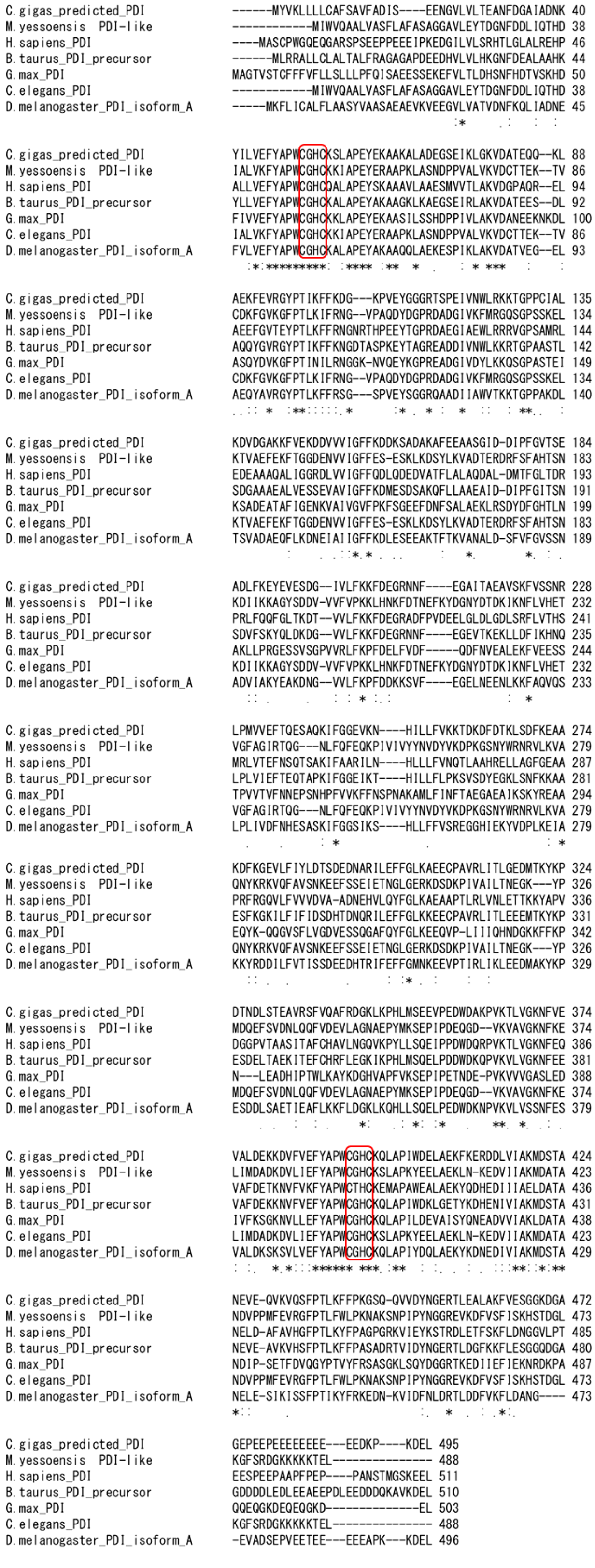

**Fig. S4**

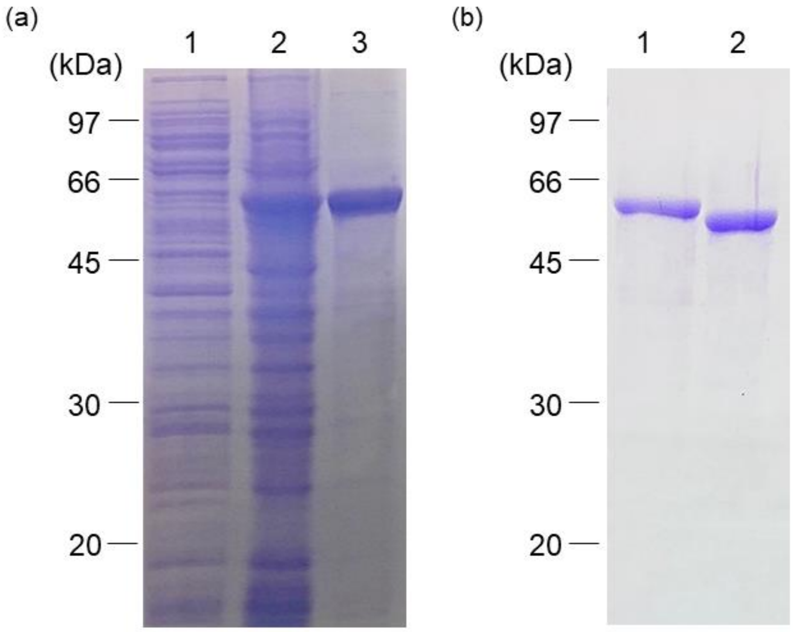


**Fig. S5**

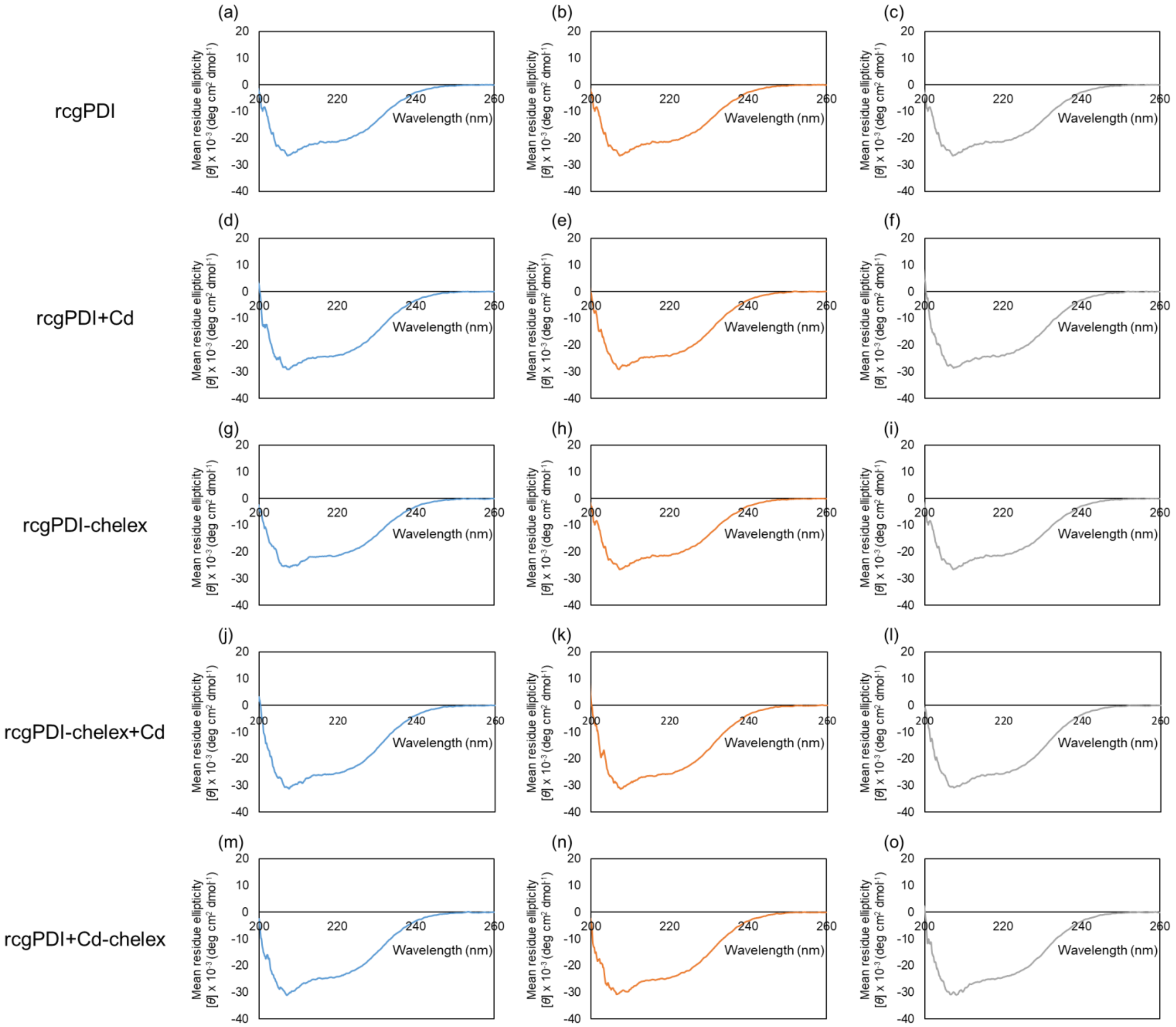

(a)
(b)
(c)
rcgPDI
(d)
(e)
(f)
rcgPDI+Cd
(g)
(h)
(i)
rcgPDI-chelex
(j)
(k)
(l)
rcgPDI-chelex+Cd
(m)
(n)
(o)
rcgPDI+Cd-chelex
Mean residue ellipticity
[θ] x 10-3 (deg cm2 dmol-1)
Wavelength (nm)
20
10
0
-10
-20
-30
-40
200
220
240
260


**Fig. S6**

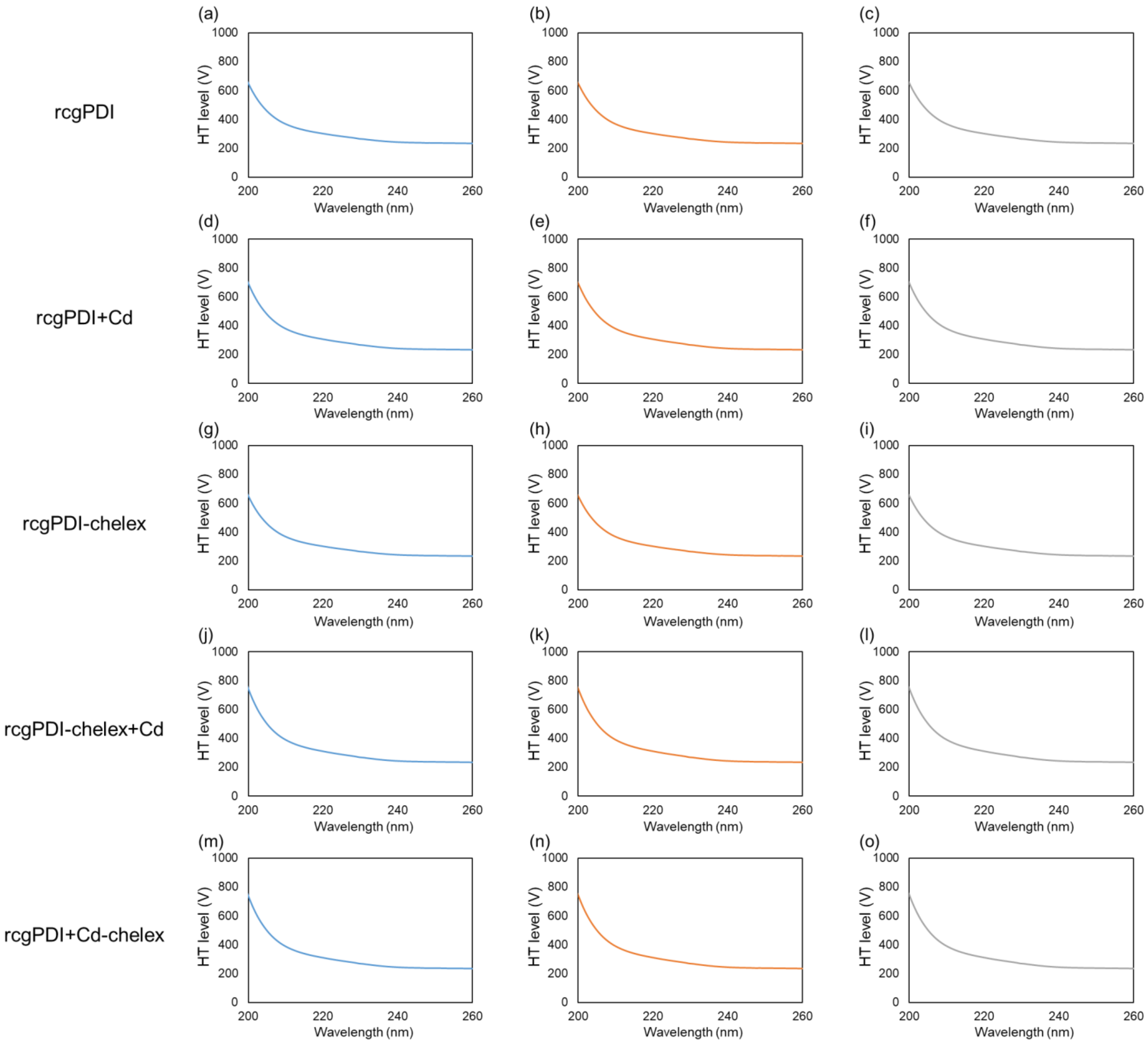
rcgPDI
rcgPDI+Cd
rcgPDI-chelex
rcgPDI-chelex+Cd
rcgPDI+Cd-chelex
(a)
(b)
(c)
(d)
(e)
(f)
(g)
(h)
(i)
(j)
(k)
(l)
(m)
(n)
(o)
HT level (V)
Wavelength (nm)
0
200
400
600
800
1000
200
220
240
260

**Fig. S7**

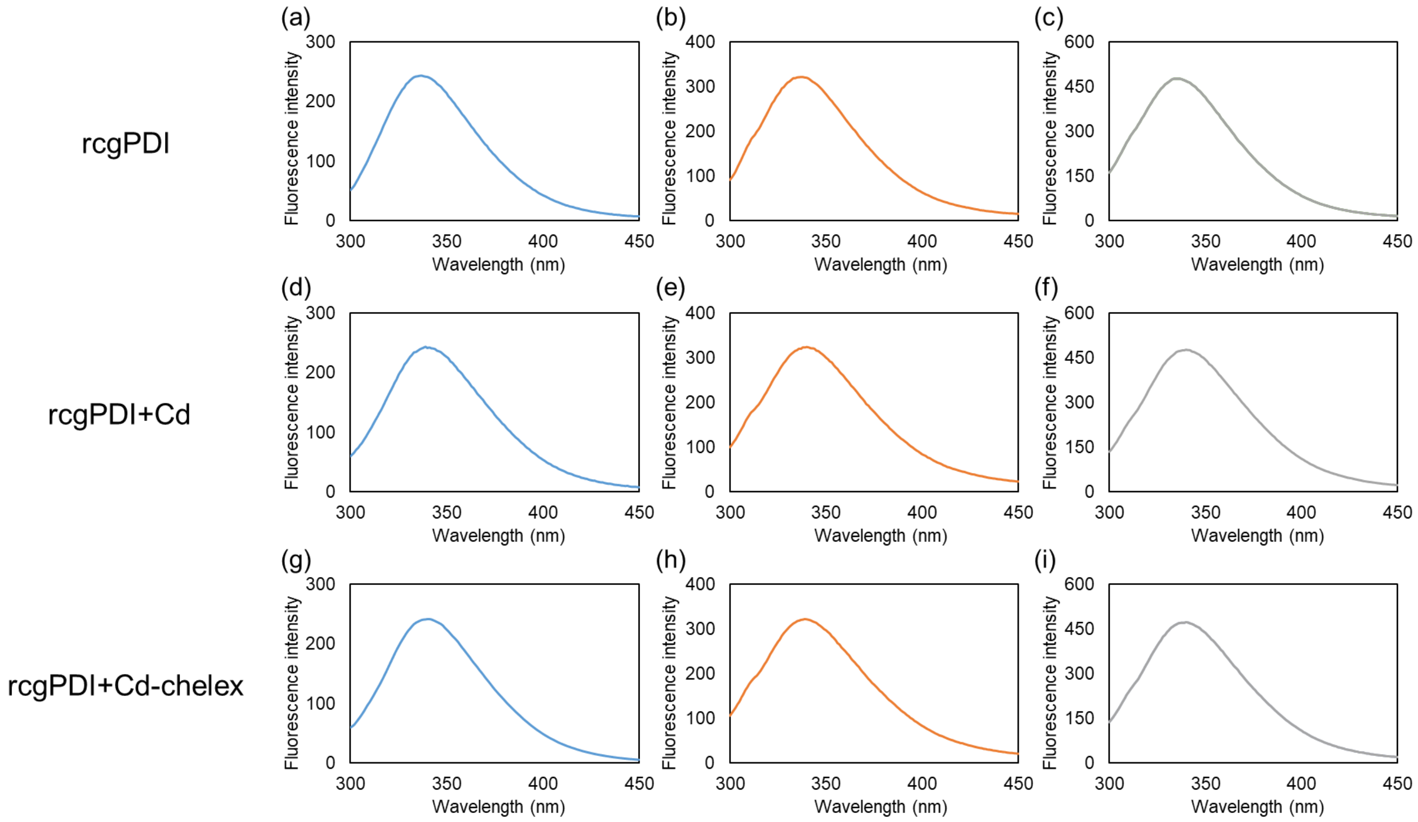
rcgPDI
rcgPDI+Cd
rcgPDI+Cd-chelex
(a)
(b)
(c)
(d)
(e)
(f)
(g)
(h)
(i)
Fluorescence intensity
Wavelength (nm)
0
100
200
300
400
150
450
600
350


**Fig. S8**

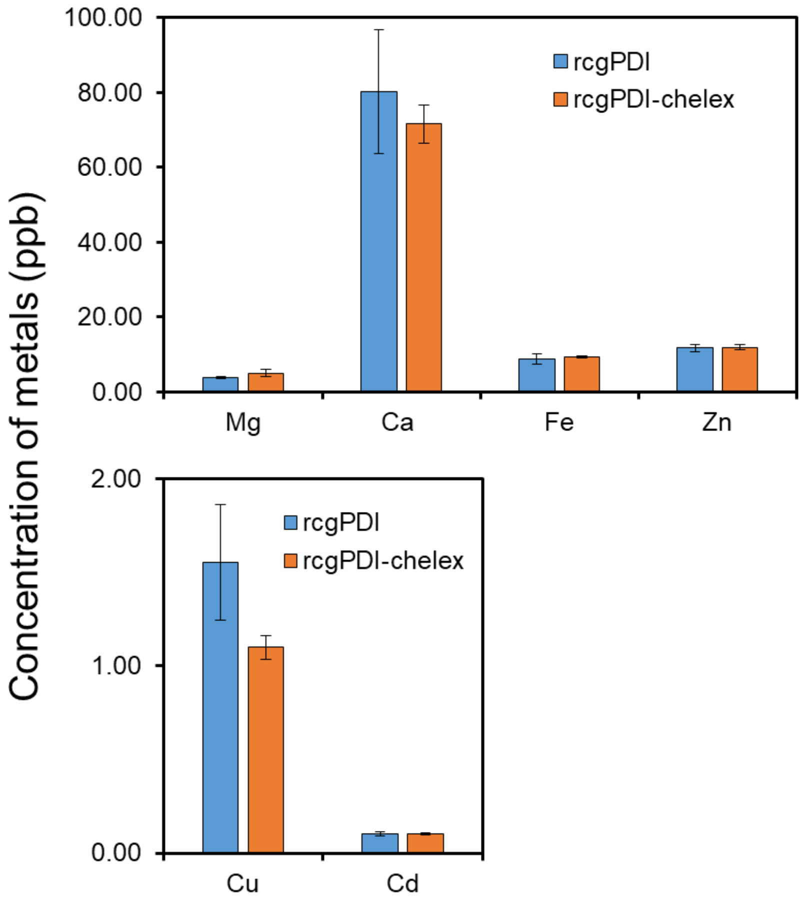


**Fig. S9**

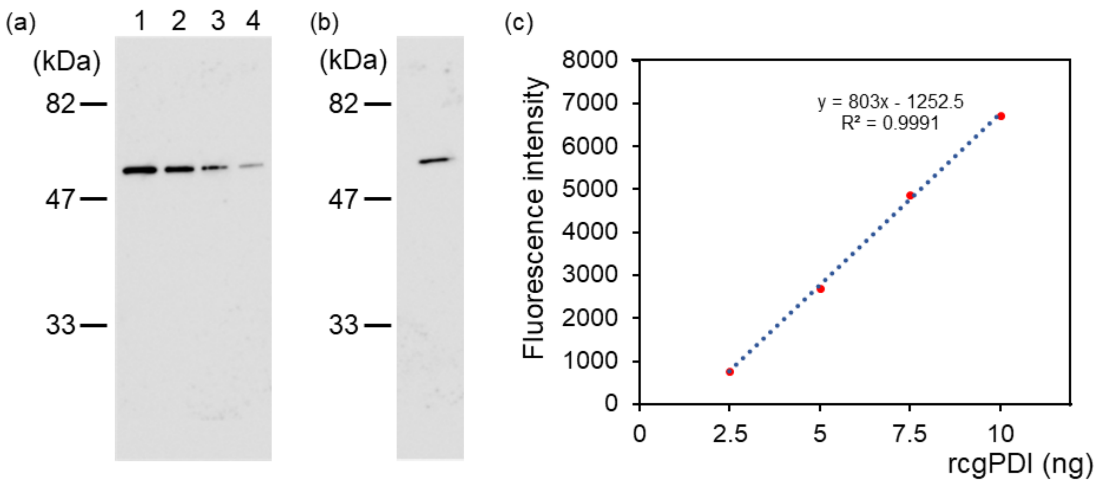


**Fig. S10**

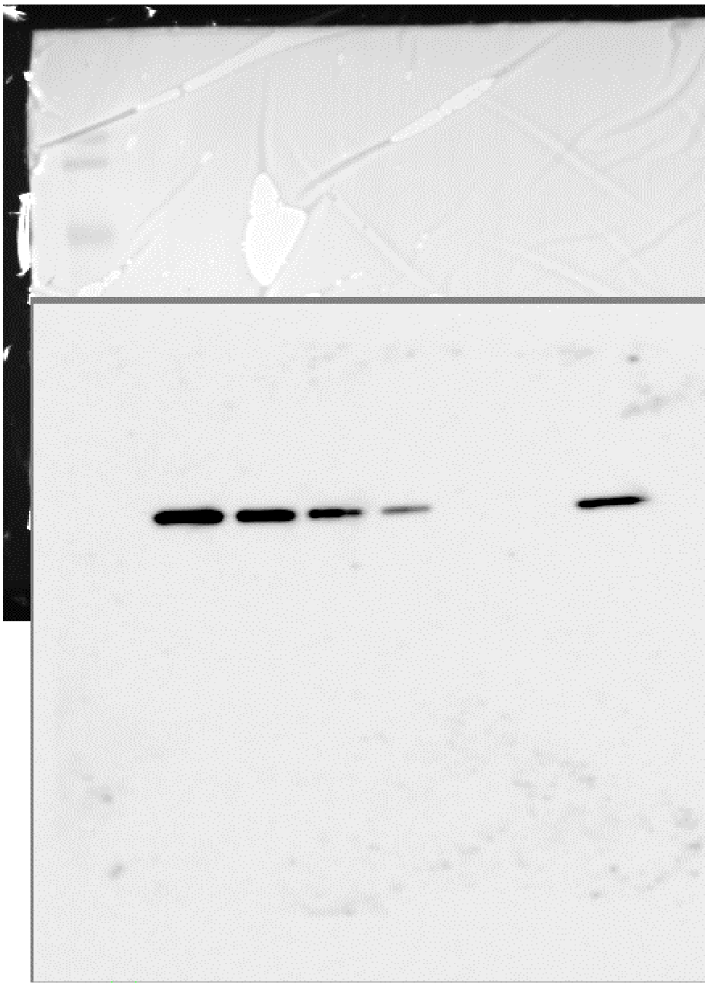

**Fig. S11**

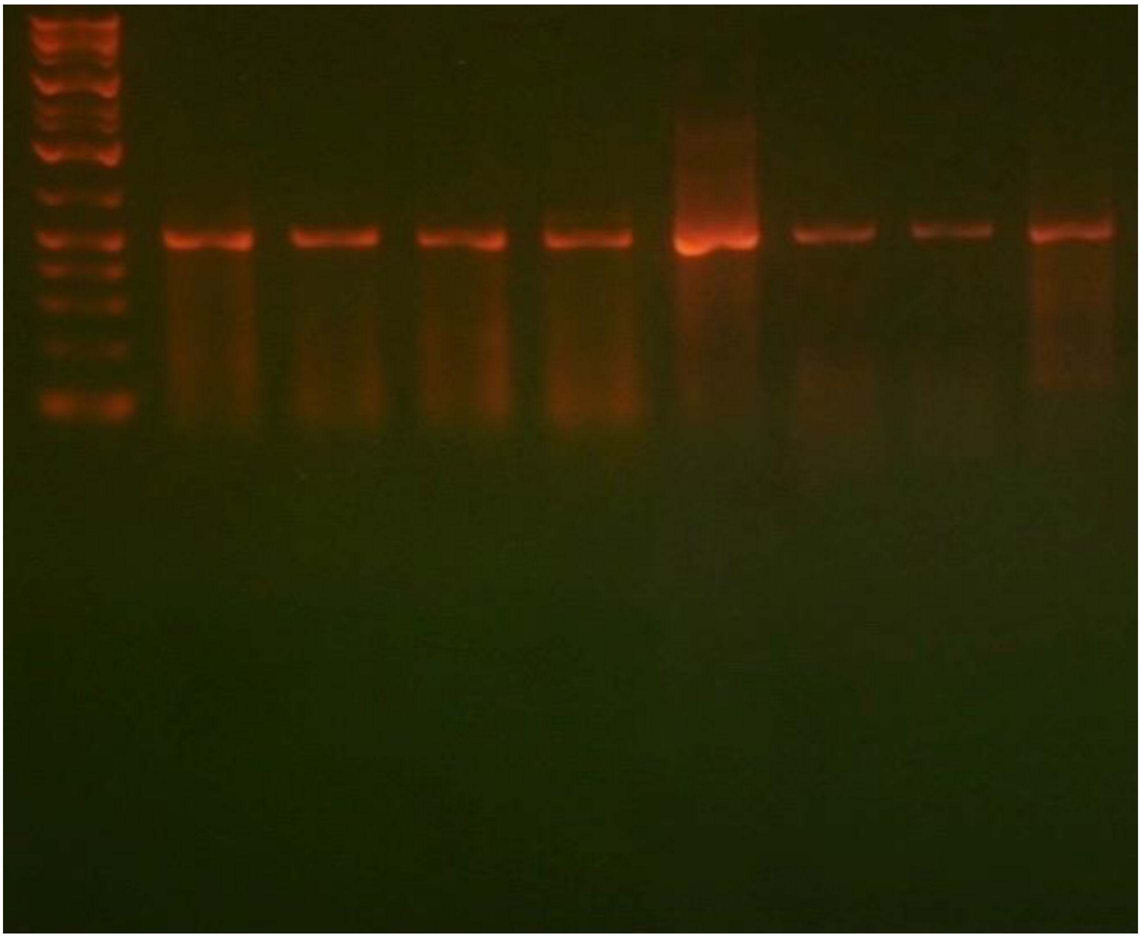

**Fig. S12**
